# Genetic risk of psychopathology predicts environmental risk

**DOI:** 10.1038/s41537-026-00805-3

**Published:** 2026-10-06

**Authors:** Adam Socrates, Jessie R. Baldwin, Robin M. Murray, Paul F. O’Reilly, Jean-Baptiste Pingault

**Affiliations:** 1 SGDP Centre, Institute of Psychiatry, Psychology and Neuroscience, King’s College London, London, UK; 2 Icahn School of Medicine at Mount Sinai, New York, NY USA; 3 Department of Clinical, Educational and Health Psychology, Division of Psychology and Language Sciences, University College London, London, UK; 4 Department of Psychosis Studies, Institute of Psychiatry, Psychology and Neuroscience, King’s College London, London, UK

**Keywords:** Human behaviour, Psychosis

## Abstract

The exposome has been conceptualised as the sum of environmental exposures across the life course, but these exposures are unlikely to be independent of genetic influences. We tested whether nine polygenic scores for psychiatric, cognitive, personality and comparison traits were associated with 49 environmental risk factors in 307,493 UK Biobank participants. Across 441 models, 212 associations remained significant after false discovery rate correction. ADHD, educational attainment, major depression, schizophrenia and bipolar disorder polygenic scores showed the broadest profiles, but the pattern of association differed substantially across traits. Schizophrenia and bipolar disorder polygenic scores were most strongly associated with interpersonal adversity and victimisation, major depression and ADHD with perception/subjectivity, and educational attainment with socioeconomic circumstances, health and health behaviour. Schizophrenia, major depression, ADHD, bipolar disorder and neuroticism polygenic scores were also more strongly associated with perception/subjectivity factors than with objective/non-perception factors. These findings show that genetic liability is associated with a broad but structured portion of the psychiatric exposome, spanning social adversity, behaviour, socioeconomic circumstances and subjective experience. They further suggest that the widespread influence of genetics on the exposome may partly reflect genetically influenced perception of environmental risk as well as differences in environmental exposure.

## Introduction

The exposome is defined as the accumulation of environmental exposures over the life course, complementary to the biological influence of the genome^1^. Although tracking the entire exposome of an individual is not currently possible, considering multiple exposures together may be particularly valuable for complex psychiatric disorders, where risk is distributed across social, developmental, behavioural, neighbourhood and health-related factors^2,3^. Environmental risk factors associated with schizophrenia and broader psychopathology include childhood adversity and trauma, cannabis use, smoking, socioeconomic disadvantage, urbanicity, migration, social isolation, pollution, poor physical health and stressful life events^4–11^. Exposome scores combining factors such as cannabis use, bullying, emotional abuse and neglect have consequently been developed to represent cumulative environmental liability to psychosis and have been associated with schizophrenia diagnosis, psychotic experiences and other clinical outcomes^8–10^.

However, ascertaining the ‘non-genetic drivers of health and disease’ in complex psychiatric disorders presents serious challenges^2,3,11^. The exposome is a dense and interrelated network rather than a set of independent exposures external to the individual. Environmental circumstances are correlated with behaviour, social position, cognition and personality, and many measures conventionally treated as environmental are themselves partly heritable, including stressful life events, social relationships, smoking, substance use, education, socioeconomic circumstances and health behaviours^12–18^. These observations reflect gene-environment correlation, whereby genetically influenced characteristics contribute to differential exposure to, selection into, modification of or responses from the environment. Small initial differences in behaviour or liability may therefore accumulate through repeated transactions between individuals and their social environments^18^.

Recent molecular-genetic evidence indicates that this issue is directly relevant to psychosis. A systematic review and meta-analysis found a statistically significant but very small association between schizophrenia polygenic liability and childhood adversity (r = 0.02), indicating that inherited liability could explain only a small proportion of the established association between adversity and psychosis^19^. Evidence of gene-environment correlation does not imply that adversity is genetically determined or that environmental effects are absent. Quasi-experimental evidence similarly indicates that childhood maltreatment remains associated with psychopathology after reducing genetic and familial confounding, although estimated effects are smaller than in conventional observational studies^20^.

Polygenic associations may also vary according to the environmental or behavioural phenotype examined. Psychiatric polygenic scores show distinct associations across dimensions of social behaviour, with effects differing by psychiatric liability, age, reporter and the particular social trait assessed^21^. Environmental risk factors may therefore capture several processes simultaneously: harmful external exposure, genetically influenced selection or evocation, familial transmission, early behavioural or affective manifestations, socioeconomic context and differences in reporting or appraisal.

A further distinction concerns environmental measures that depend strongly on perception, memory and social interpretation. Genetic liability for psychopathology may influence attention to threat, affective processing, recall, rejection sensitivity or the interpretation of interpersonal experience^22,23^. Individuals with greater liability to depression or psychosis may therefore experience or report apparently similar circumstances differently. Consistent with this possibility, neuroticism polygenic liability has been associated with increases in perceived daily stress during a prolonged stress period^24^.

This does not make subjective reports less valid or clinically important. Rather, their psychological meaning may be part of the pathway through which environmental experiences become associated with psychopathology. Subjective reports may incorporate cognitive bias or mood-congruent recall, but may also capture threat, humiliation, rejection, lack of safety or emotional meaning that is not represented by externally recorded events or simple behavioural indicators.

Evidence from childhood adversity research illustrates this distinction. Prospective or externally documented adversity and retrospective subjective reports identify substantially different groups, suggesting that they are related but not interchangeable measures^25,26^. A 2023 meta-analysis found that subjective adversity was more strongly associated with psychopathology than objective adversity, while agreement between the two forms of measurement was limited^26^. A subsequent systematic review and meta-analysis of 24 studies and 15,485 individuals found that retrospective maltreatment measures were more strongly associated with psychopathology than prospective measures; after accounting for their interdependence, the retrospective association was 69% larger^27^. Related longitudinal evidence indicates that the subjective experience of childhood maltreatment is particularly associated with the persistence of emotional disorders, although continuity in psychopathology explains part of this relationship^28^.

This distinction may be particularly important for social and interpersonal risk factors. Loneliness and perceived hostility are not equivalent to infrequent social contact, and dissatisfaction with close relationships is not equivalent to having few interactions with friends or relatives. Behavioural measures capture participation or exposure, whereas subjective measures additionally capture the experienced quality, emotional meaning and perceived safety of the social environment. Depression polygenic risk has also been examined jointly with social support under stress, indicating that genomic risk may modify the relationship between support and depressive symptoms^29^.

This issue is central to exposome research because pooled indices may combine externally imposed exposures, behavioural outcomes, socioeconomic consequences, interpersonal experiences and perception/subjectivity measures that differ substantially in their relationship with inherited liability.

Here, we systematically examined whether genetic liability to psychiatric, psychological, cognitive and comparison traits was associated with a measured section of the psychiatric exposome. In 307,493 UK Biobank participants without recorded schizophrenia, bipolar disorder or major depressive disorder diagnoses or antipsychotic medication use, we tested polygenic scores for schizophrenia, bipolar disorder, major depression, ADHD, educational attainment, neuroticism, extraversion, Alzheimer’s disease and LDL cholesterol against 49 environmental risk factors. ADHD was included as a neurodevelopmental liability potentially relevant to behavioural, educational, occupational, financial and familial environments; educational attainment and personality scores examined cognitive, socioeconomic, affective and social pathways; and Alzheimer’s disease and LDL cholesterol were non-psychiatric comparison traits.

Rather than treating environmental risk as a single construct, we examined individual associations and compared five categories distinguishing perception/subjectivity, interpersonal adversity and victimisation, objective-life events, objective-socioeconomic and physical-environmental circumstances, and behavioural or lifestyle factors. We additionally compared the seven perception/subjectivity measures with 25 objective-life-event and objective-socioeconomic/environmental measures, excluding interpersonal victimisation and behavioural/lifestyle factors from both sides of this contrast. Four matched comparisons covered similar aspects of health, finances, social connection and family relationships.

We hypothesised that polygenic liability for psychiatric disorders would be associated with multiple environmental risk factors; that the pattern and magnitude of these associations would differ across polygenic scores and environmental-risk categories; and that schizophrenia and major depression polygenic scores would be more strongly associated with perception/subjectivity measures than with comparatively objective/non-perception measures.

## Results

### Genetic influences on individual risk factors of the psychiatric exposome

**Fig. 1:**
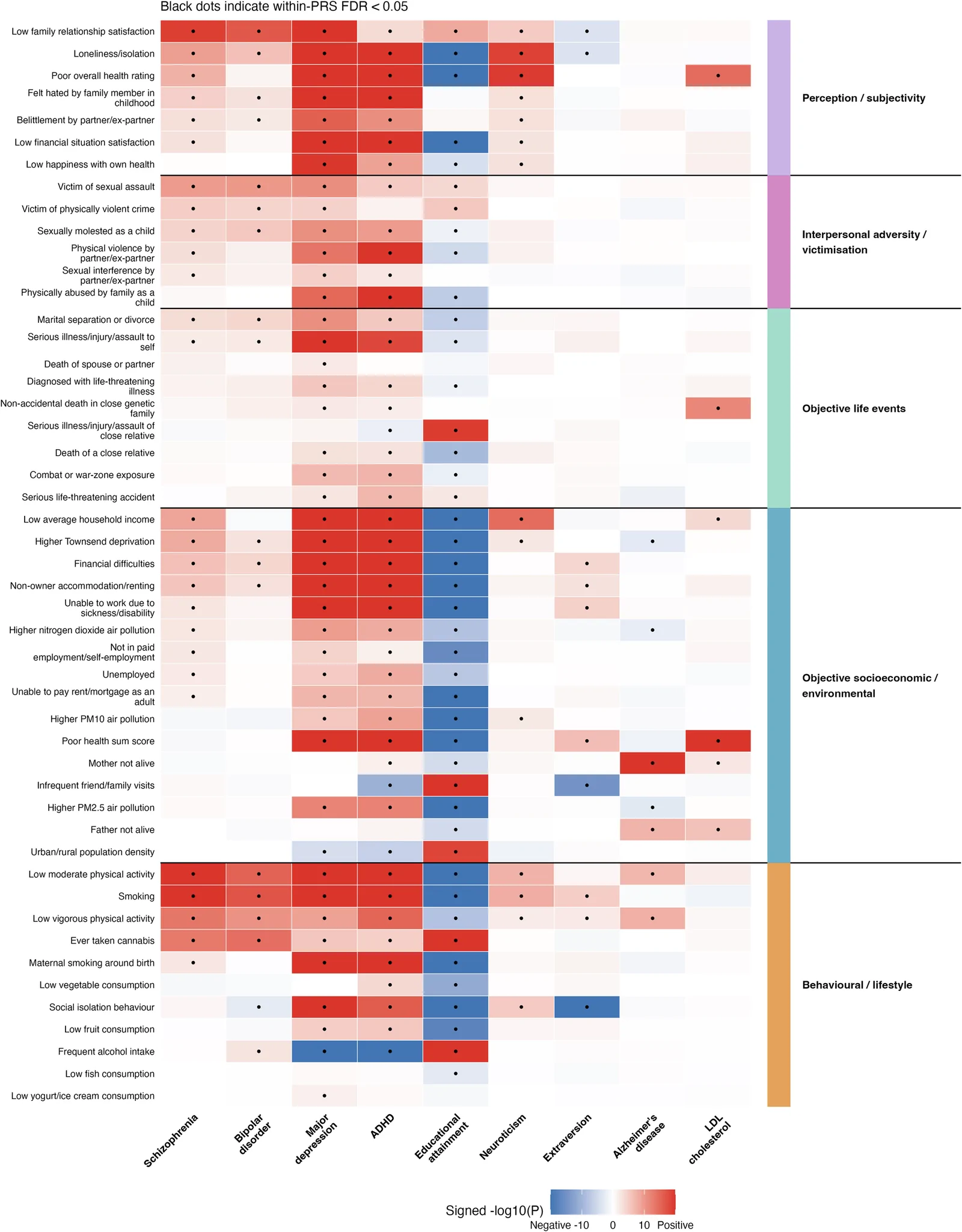
Associations between polygenic scores and 49 environmental risk factors. Cell colour shows the signed −log10(P) from standardised linear models; red denotes positive associations with higher coded risk and blue denotes negative associations. Black dots indicate within-PRS FDR < 0.05. Coloured side bars identify the five ERF categories.

Across the nine PRS and 49 environmental risk factors (ERFs), 212 of 441 associations survived FDR correction within each PRS. ADHD PRS was associated with 44 ERFs, educational attainment PRS with 43, major depression PRS with 43, schizophrenia PRS with 27, bipolar disorder PRS with 18, neuroticism PRS with 14, extraversion PRS with 10, Alzheimer’s disease PRS with seven, and LDL cholesterol PRS with six (Fig. 1). Analytic sample sizes ranged from 43,333 to 304,634 across models.

### Schizophrenia PRS

Schizophrenia PRS predicted several established ERFs for schizophrenia, including cannabis use (*β* = 0.024, *P* = 6.45 × 10⁻¹⁴), smoking (*β* = 0.018, *P* = 5.34 × 10⁻²³), higher Townsend deprivation (*β* = 0.010, *P* = 5.97 × 10⁻⁹), sexual assault (*β* = 0.022, *P* = 1.62 × 10⁻¹⁰) and violent victimisation (*β* = 0.015, *P* = 1.40 × 10⁻⁵). These findings extend previous evidence that schizophrenia polygenic liability is associated with childhood adversity and cannabis involvement, although reported gene-environment correlations are generally small^19,30–32^.

More unexpectedly, the largest effect estimate was for low family relationship satisfaction (*β* = 0.029, *P* = 6.68 × 10⁻²⁰), while low moderate physical activity provided the strongest statistical evidence (*β* = 0.022, *P* = 9.89 × 10⁻³⁴). Schizophrenia PRS was also associated with loneliness, feeling hated by a family member during childhood, partner violence and belittlement, low vigorous physical activity, lower income and housing disadvantage. In contrast, there was little evidence of association with frequency of family or friend visits, serious accidents, bereavement, combat exposure, or most family-health events. The distinction between low relationship satisfaction and actual frequency of social contact suggests that schizophrenia liability was related more strongly to the experienced quality of close relationships than to the quantity of social contact.

The concentration of associations in cannabis use, smoking, victimisation, deprivation and adverse relationships is compatible with gene-environment correlation, but does not indicate that these exposures are reducible to genetic liability. Previous studies have found shared genetic liability between schizophrenia and cannabis use alongside associations between cannabis exposure and psychotic experiences that persist after adjustment for schizophrenia PRS^30–32^. Similarly, the meta-analytic association between schizophrenia PRS and childhood adversity is small, indicating that inherited liability explains only a limited proportion of the established adversity-psychosis association^19^.

Particulate-matter findings were inconsistent: higher PM2.5 and nitrogen dioxide exposure were associated with schizophrenia PRS, whereas PM10 was not. This does not support a simple distinction in which physical environmental exposures are wholly independent of genetic liability, but neither does it indicate a consistent schizophrenia polygenic association across air-pollution measures. Individual schizophrenia PRS estimates across all 49 ERFs are shown in Supplementary Fig. 2.

### Major depression PRS

Major depression PRS was associated with 43 ERFs. Its strongest associations were with poor overall health rating (*β* = 0.052, *P* = 4.65 × 10⁻¹⁸⁴), smoking (*β* = 0.045, *P* = 2.47 × 10⁻¹³⁸), loneliness (*β* = 0.044, *P* = 3.88 ×  10⁻¹²⁹), low financial satisfaction (*β* = 0.043, *P* = 4.31 × 10⁻⁴³) and low family relationship satisfaction (*β* = 0.040, *P* = 4.73 × 10⁻³⁷). Associations were also observed with lower household income, financial difficulties, deprivation, inability to work because of sickness or disability, housing disadvantage, childhood adversity and adverse treatment by partners.

The profile was therefore not restricted to negative perception. Major depression liability was associated with objective-socioeconomic disadvantage and health impairment as well as loneliness and dissatisfaction with health, finances and relationships. Nevertheless, the particularly strong associations with perceived health and social circumstances suggest that genetically influenced affective and cognitive processes may contribute to how environmental circumstances are experienced, alongside effects on exposure to adversity^22,23,25,28^.

### ADHD PRS

ADHD PRS showed the widest association profile, with 44 of 49 ERFs surviving correction. The largest associations were with smoking (*β* = 0.057, *P* = 1.00 × 10⁻²²⁰), poor overall health rating (*β* = 0.048, *P* = 1.49 × 10⁻¹⁵⁶), lower household income (*β* = 0.043, *P* = 1.45 × 10⁻¹³⁰), childhood physical abuse (*β* = 0.048, *P* = 2.36 × 10⁻⁴⁴) and maternal smoking around birth (*β* = 0.042, *P* = 5.07 × 10⁻¹⁰⁵). Associations extended across health behaviour, interpersonal adversity, physical health, employment, housing, deprivation and financial circumstances.

The breadth of the ADHD profile was unlikely to reflect greater discovery-GWAS power, because the ADHD GWAS was smaller and identified fewer genome-wide significant loci than the schizophrenia and major depression GWAS^33–35^. Rather, it is consistent with the wide functional correlates of ADHD liability: impulsivity and impaired self-regulation may contribute to smoking and other health behaviours; attentional and executive difficulties may influence education, employment and financial stability; and associations with maternal smoking and childhood circumstances may partly reflect passive gene-environment correlation. The genetic correlations of ADHD with smoking, educational outcomes and other behavioural traits support effects across several developmental pathways rather than one specific exposure class^35^.

### Bipolar disorder PRS

Bipolar disorder PRS was associated with 18 ERFs. The strongest associations were with cannabis use (*β* = 0.026, *P* = 6.14 × 10⁻¹⁵), low family relationship satisfaction (*β* = 0.027, P = 2.94 × 10⁻¹⁷), sexual assault (*β* = 0.022, *P* = 8.12 × 10⁻¹¹), smoking (*β* = 0.016, *P* = 1.06 × 10⁻¹⁷) and low moderate physical activity (*β* = 0.015, *P* = 4.01 × 10⁻¹⁶). Further associations with childhood sexual abuse, violent victimisation, partner adversity and loneliness produced substantial overlap with the schizophrenia profile, particularly for cannabis use and interpersonal adversity, consistent with the shared genetic liability between schizophrenia and bipolar disorder^36^.

### Educational attainment PRS

Educational attainment PRS was associated with 43 ERFs and generally showed the opposite direction to the psychiatric PRS. Higher educational attainment PRS was associated with higher household income, better health, lower deprivation, home ownership, employment, lower smoking, and lower maternal smoking exposure. The largest inverse associations were with low household income (*β* = −0.094, *P* < 1 × 10⁻³⁰⁰), poor overall health rating (*β* = −0.068, *P* = 1.27 × 10⁻³⁰⁰), smoking (*β* = −0.051, *P* = 1.87 × 10⁻¹⁷⁴) and maternal smoking around birth (*β* = −0.045, *P* = 9.57 × 10⁻¹¹⁷).

However, higher educational attainment PRS was also associated with more frequent alcohol intake (*β* = 0.055, *P* = 2.76 × 10⁻²⁰⁶) and cannabis use (*β* = 0.041, *P* = 2.06 × 10⁻³⁴), while its pooled association with interpersonal adversity/victimisation was close to zero and not statistically significant. Educational attainment liability was therefore strongly related to socioeconomic position, health and health behaviour, but not to uniformly lower exposure to substance use, abuse, assault or adverse relationships^13,37^.

### Personality and comparison PRS

Neuroticism PRS was associated with 14 ERFs, with the strongest associations involving loneliness, poor perceived health, low physical activity, lower income and behavioural social isolation. Extraversion PRS was inversely associated with behavioural social isolation, infrequent contact with friends or family, loneliness and low family relationship satisfaction. The parallel extraversion associations with subjective loneliness and behavioural social contact indicate that the social findings reflected both experienced disconnection and differences in social participation.

Alzheimer’s disease and LDL cholesterol PRS showed fewer and more restricted associations and did not reproduce the extensive interpersonal, behavioural and socioeconomic patterns observed for the psychiatric, personality and educational attainment PRS.

### Genetic influences across environmental risk-factor categories

**Fig. 2:**
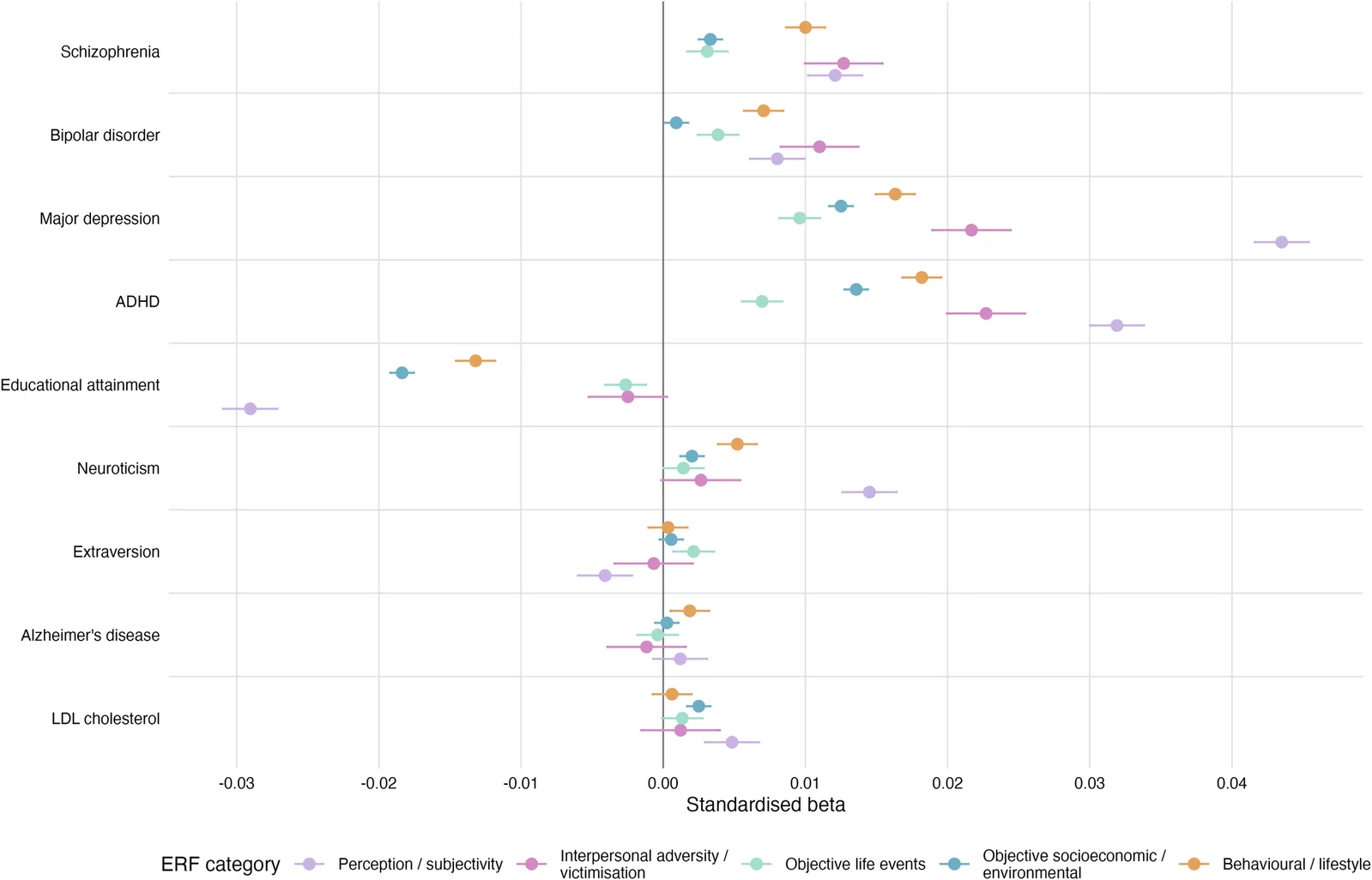
Inverse-variance pooled standardised associations across five conceptually defined ERF categories. Points show pooled *β* estimates and horizontal lines show 95% confidence intervals.

Figure 2 summarises the final five-category analysis. Schizophrenia PRS showed its largest pooled association with interpersonal adversity/victimisation (*β* = 0.013, 95% CI 0.010–0.015), followed by perception/subjectivity (*β* = 0.012, 95% CI 0.010–0.014) and behavioural/lifestyle factors (*β* = 0.010, 95% CI 0.009–0.011). Associations with objective-socioeconomic/environmental factors (*β* = 0.003) and objective-life events (*β* = 0.003) were smaller.

Bipolar disorder PRS followed a similar pattern, with the largest pooled association for interpersonal adversity/victimisation (*β* = 0.011, 95% CI 0.008–0.014), followed by perception/subjectivity (*β* = 0.008) and behavioural/lifestyle factors (*β* = 0.007).

Major depression and ADHD PRS showed their largest pooled associations with perception/subjectivity. For major depression, the pooled estimates were *β* = 0.043 for perception/subjectivity, *β* = 0.022 for interpersonal adversity/victimisation, *β* = 0.016 for behavioural/lifestyle factors, *β* = 0.013 for objective-socioeconomic/environmental factors and *β* = 0.010 for objective-life events. The corresponding ADHD estimates were *β* = 0.032, 0.023, 0.018, 0.014, and 0.007.

Neuroticism PRS was concentrated in perception/subjectivity (*β* = 0.015), whereas its interpersonal adversity/victimisation estimate was small (*β* = 0.003). Educational attainment PRS showed its largest inverse associations with perception/subjectivity (*β* = −0.029) and objective-socioeconomic/environmental factors (*β* = −0.018); its pooled association with interpersonal adversity/victimisation was close to zero (*β* = −0.002). Pairwise contrasts between the five categories are shown in Supplementary Fig. 1.

### Perception/subjectivity versus objective/non-perception factors

**Fig. 3:**
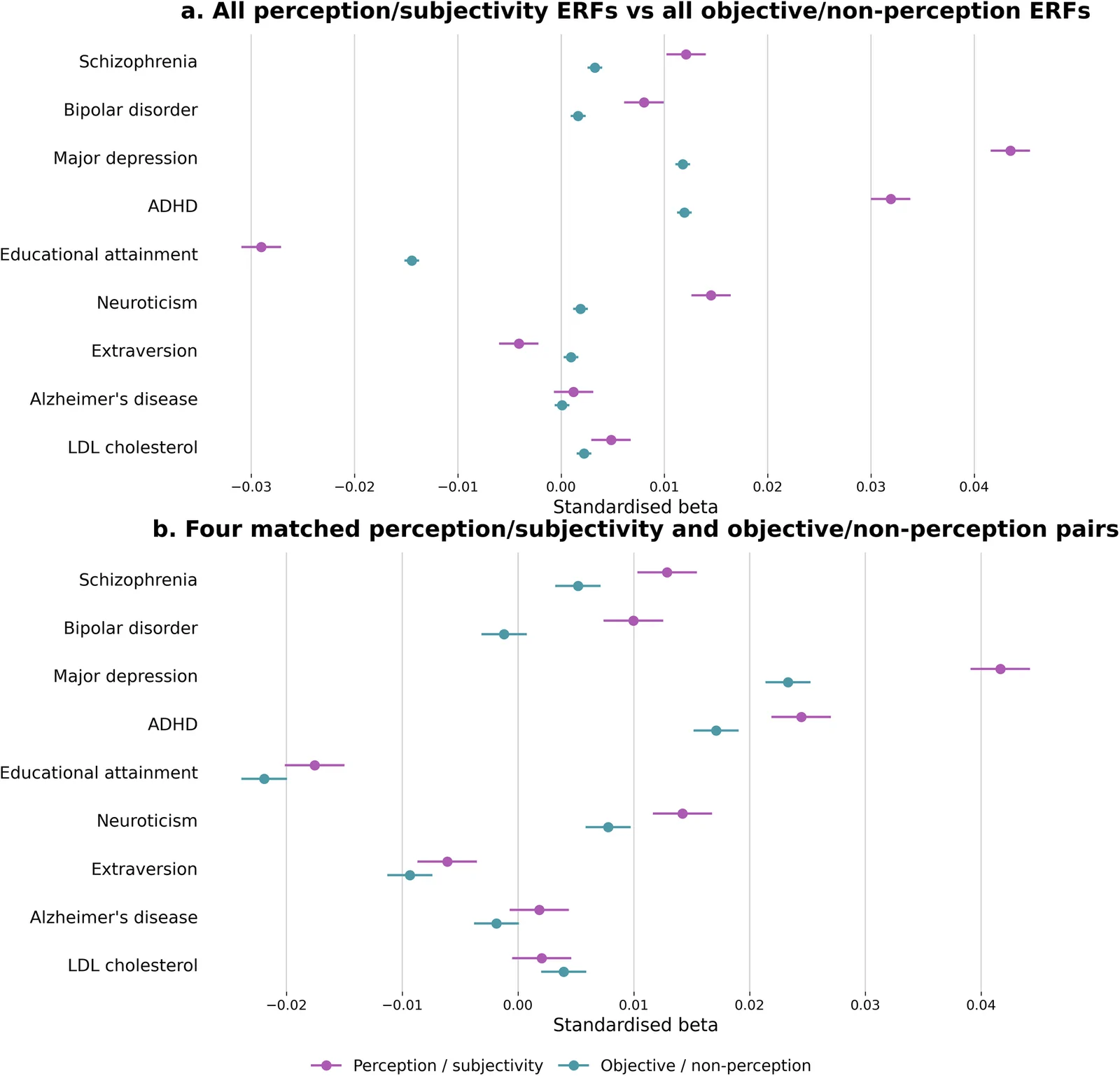
Perception/subjectivity and objective/non-perception comparisons. **a** compares all seven perception/subjectivity ERFs with 25 objective-life-event and objective-socioeconomic/environmental ERFs. **b** compares four conceptually matched perception/subjectivity and objective/non-perception sets. Colours distinguish the two comparison groups; points show pooled *β* estimates and horizontal lines show 95% confidence intervals.

Schizophrenia PRS showed a stronger pooled association with the seven perception/subjectivity measures (*β* = 0.012, 95% CI 0.010–0.014) than with the 25 objective/non-perception measures (*β* = 0.003, 95% CI 0.003–0.004; difference *P* = 1.58 × 10⁻¹⁷; Fig. 3a).

Bipolar disorder PRS showed a stronger pooled association with the seven perception/subjectivity measures (*β* = 0.008, 95% CI 0.006–0.010) than with the 25 objective/non-perception measures (*β* = 0.002, 95% CI 0.001–0.002; difference *P* = 9.54 × 10⁻¹⁰; Fig. 3a).

Major depression PRS showed a stronger pooled association with the seven perception/subjectivity measures (*β* = 0.043, 95% CI 0.042-0.045) than with the 25 objective/non-perception measures (*β* = 0.012, 95% CI 0.011–0.012; difference *P* = 6.62 × 10⁻²⁰⁵; Fig. 3a).

ADHD PRS showed a stronger pooled association with the seven perception/subjectivity measures (*β* = 0.032, 95% CI 0.030–0.034) than with the 25 objective/non-perception measures (*β* = 0.012, 95% CI 0.011–0.013; difference *P* = 2.60 × 10⁻⁸³; Fig. 3a).

Neuroticism PRS showed a stronger pooled association with the seven perception/subjectivity measures (*β* = 0.015, 95% CI 0.013–0.016) than with the 25 objective/non-perception measures (*β* = 0.002, 95% CI 0.001–0.003; difference *P* = 3.35 × 10⁻³⁴; Fig. 3a).

The psychiatric scores were also associated with objective/non-perception factors. Their larger perception/subjectivity estimates therefore indicate an additional contribution from perception, affect, social interpretation, or reporting rather than showing that the associations were attributable only to subjective measurement. Educational attainment PRS was inversely associated with both groups, with a stronger inverse association for perception/subjectivity (*β* = −0.029) than objective/non-perception factors (*β* = −0.014; difference *P* = 2.49 × 10⁻⁴⁴). Interpersonal adversity/victimisation and behavioural/lifestyle factors were excluded from this comparison.

### Matched perception/subjectivity and objective/non-perception comparisons

Schizophrenia PRS: perception/subjectivity *β* = 0.013 (95% CI 0.010–0.015) and objective/non-perception *β* = 0.005 (95% CI 0.003–0.007; difference *P* = 2.83 × 10⁻⁶, FDR *q* = 8.50 × 10⁻⁶).

Major depression PRS: perception/subjectivity *β* = 0.042 (95% CI 0.039–0.044) and objective/non-perception *β* = 0.023 (95% CI 0.021-0.025; difference *P* = 6.63 × 10⁻²⁹, FDR *q* = 5.97 × 10⁻²⁸).

ADHD PRS: perception/subjectivity *β* = 0.024 (95% CI 0.022–0.027) and objective/non-perception β = 0.017 (95% CI 0.015–0.019; difference *P* = 7.63 × 10⁻⁶, FDR *q* = 1.72 × 10⁻⁵).

Bipolar disorder PRS: perception/subjectivity *β* = 0.010 (95% CI 0.007–0.013) and objective/non-perception *β* = −0.001 (95% CI −0.003–0.001; difference *P* = 1.33 × 10⁻¹¹, FDR *q* = 6.00 × 10⁻¹¹).

Neuroticism PRS: perception/subjectivity *β* = 0.014 (95% CI 0.012–0.017) and objective/non-perception *β* = 0.008 (95% CI 0.006–0.010; difference *P* = 8.84 × 10⁻⁵, FDR *q* = 1.59 × 10⁻⁴).

Educational attainment PRS: perception/subjectivity *β* = −0.018 (95% CI −0.020 to −0.015) and objective/non-perception *β* = −0.022 (95% CI −0.024 to −0.020; difference *P* = 0.0083, FDR *q* = 0.0124).

The clearest matched distinction concerned family relationships and social disconnection. Psychiatric PRS were generally more strongly associated with the experienced quality of family relationships than with frequency of social contact. Loneliness also exceeded behavioural social isolation for several scores, whereas health and financial pairs were less uniform. These findings support a role for perception and subjectivity but do not imply that objective circumstances are unimportant (Fig. 3b and Supplementary Table 5). Individual estimates for each of the four matched pairs are shown in Supplementary Fig. 3.

## Discussion

### Principal findings

Polygenic liability was associated with a substantial but heterogeneous proportion of the environmental, behavioural and subjective risk factors examined in participants without recorded schizophrenia, bipolar disorder or major depressive disorder diagnoses or antipsychotic medication use. Across 441 models, 212 associations survived within-score FDR correction. Schizophrenia and bipolar disorder showed their largest pooled associations with interpersonal adversity and victimisation; major depression and ADHD with perception/subjectivity; and educational attainment with socioeconomic circumstances, health and health behaviour. Alzheimer’s disease and LDL cholesterol showed fewer and more restricted associations.

These findings extend evidence for the ‘paradox of heritable environments’, whereby experiences conventionally treated as environmental are partly associated with inherited characteristics^12–18^. They do not establish direction or mechanism. Associations may reflect active, passive or evocative gene-environment correlation; indirect pathways through behaviour, education, health or socioeconomic position; early manifestations of liability; appraisal and recall; or reciprocal processes over time. The small meta-analytic association between schizophrenia polygenic liability and childhood adversity similarly indicates detectable gene-environment correlation but suggests that it explains little of the established adversity-psychosis relationship^19^.

### Schizophrenia liability and environmental risk

Schizophrenia polygenic score was associated with several established environmental risk factors for psychosis, including cannabis use, smoking, deprivation, sexual assault and violent victimisation^4–11,38,39^. Associations were also observed with low physical activity, lower income, housing disadvantage and adverse family or partner relationships. The concentration of findings in substance use, interpersonal adversity, social experience and socioeconomic conditions suggests several routes through which schizophrenia liability may become correlated with measured environmental risk rather than a single general adversity pathway.

Some associations may involve behavioural selection related to motivation, cognition, reward processing or social functioning; others may involve social responses to genetically influenced behaviour, shared familial liabilities or cumulative transactions between individuals and their environments^18,19,40,41^. Associations with maltreatment, assault and victimisation require a multifactorial interpretation. They do not imply that victims cause or are responsible for abusive treatment, nor that such experiences are genetically determined. Associations could reflect familial transmission, unsafe social or neighbourhood contexts, selection or evocation, perpetrator and contextual characteristics, and differences in memory or appraisal. Prospective or documented adversity and retrospective reports identify partly different groups^25–27^, while quasi-experimental evidence indicates that maltreatment remains associated with mental-health outcomes after reducing genetic and familial confounding^20^. These findings indicate complexity in the origins and measurement of victimisation, not evidence against its environmental or harmful effects.

The schizophrenia profile was selective. Schizophrenia liability was associated with nitrogen dioxide and PM2.5 but not PM10, and showed little evidence of association with diet, bereavement, serious accidents, combat exposure, frequency of contact with friends or relatives, or most family-health events. These null and inconsistent findings argue against a uniformly genetically patterned psychiatric exposome, although measurement error, power, and heterogeneous mechanisms constrain interpretation.

### Perception and subjective experience

A key finding was the stronger association of psychiatric polygenic liability with perception/subjectivity factors than with 25 objective/non-perception factors after interpersonal adversity/victimisation and behavioural/lifestyle measures were excluded. The pattern was observed for schizophrenia, major depression, ADHD, bipolar disorder and neuroticism and remained visible in the matched analysis, where subjective and comparatively objective measures covered similar aspects of social connection, health, finances and family relationships.

The social comparisons were particularly informative. Schizophrenia and major depression liability was associated with dissatisfaction with family relationships but not infrequent contact with friends or relatives. Loneliness was also more strongly associated than behavioural social isolation for several psychiatric scores. The relevant signal therefore concerned the experienced quality of relationships rather than simply frequency of interaction. Contact counts cannot establish whether relationships are supportive, rejecting, conflictual or emotionally satisfying, while loneliness can occur despite frequent social contact.

Psychiatric liability could contribute through attentional bias, interpretation of ambiguous social information, rejection sensitivity, affective processing, or memory^22,23^. Neuroticism polygenic liability has been associated with increases in perceived daily stress^24^. However, stronger genetic associations with subjective measures should not be reduced to biased reporting. Subjective measures may capture relational threat, humiliation, coercion, insecurity or emotional meaning omitted by behavioural indicators and administrative records. Comparisons of prospective and retrospective adversity similarly suggest that first-person experience is psychologically consequential rather than merely an inferior proxy^25–28^.

The pattern was not universal. Poor overall health was more strongly related than health satisfaction for several scores, and Alzheimer’s disease, extraversion and LDL cholesterol did not show consistent matched subjective effects. The psychiatric pattern is therefore unlikely to be an inevitable consequence of self-report or pooling. These analyses cannot determine whether appraisal preceded environmental difficulty, developed in response to it, or emerged reciprocally. Longitudinal and genetically informative studies with repeated subjective and objective assessments are needed.

### ADHD and contrasting pathways

ADHD polygenic score was associated with 44 of 49 factors, including smoking, maternal smoking around birth, health, childhood adversity, income, employment, housing and financial circumstances. This breadth is unlikely to be explained simply by superior discovery-GWAS power, because the ADHD GWAS was smaller and identified fewer genome-wide significant loci than the schizophrenia and major depression GWAS^33–35^. Impulsivity, altered reward processing and impaired self-regulation may contribute to smoking and other health behaviours, while attentional and executive difficulties may influence educational, occupational and financial trajectories. Associations with maternal smoking and childhood circumstances may additionally reflect passive gene-environment correlation through genetically related parental traits.

The contrast between score profiles was as informative as the overall number of associations. Schizophrenia and bipolar disorder were concentrated in interpersonal adversity and victimisation, major depression and ADHD in perception/subjectivity, neuroticism in loneliness and perceived health, and educational attainment in socioeconomic and health pathways. Educational attainment was also associated with more frequent alcohol and cannabis use and therefore did not operate as a general protective score. These differences support retaining individual factors as the primary level of analysis; pooled categories clarify patterns but combine heterogeneous and correlated measures.

### Implications, strengths and limitations

The exposome is often presented as the environmental counterpart to the genome, but the two are not independent systems. Psychiatric exposome measures may include externally imposed events, inherited behavioural tendencies, socioeconomic consequences, social responses, subjective experiences and early manifestations of liability. Combining them may improve prediction, but a resulting score cannot automatically be interpreted as exogenous environmental causation. A genetically informed framework should distinguish association with exposure, association with perception or reporting, and causal contribution after genetic and familial influences are considered. This study addresses the first two questions, not the third^41^.

Strengths include the large sample, analysis of 49 factors within a common framework, updated PRS-CS scores, retention of ordinal and continuous information, comparison of nine polygenic scores, and complementary individual, category-level and matched analyses. Excluding participants with recorded major psychiatric diagnoses or antipsychotic medication use reduced the likelihood that findings were driven entirely by diagnosed disorder or treatment, although subclinical symptoms, undiagnosed disorders and previous episodes remain possible.

Polygenic scores capture only part of inherited liability and differ in predictive power and genetic architecture. The cross-sectional and partly retrospective data cannot establish temporal ordering or distinguish gene-environment correlation from downstream consequences. Many factors were self-reported, while comparatively objective measures may still involve reporting and measurement error; conversely, externally defined measures may not capture psychological meaning. Pooled analyses are descriptive summaries. Model-specific sample sizes varied substantially across ERFs, so comparisons differed in precision and power, and differences in phenotype reliability may attenuate standardised associations. The large sample provides power to detect very small effects, and the results are interpretive rather than clinically predictive. The small PRS-ERF effects are also expected given that current molecular PRS explain only a limited fraction of liability even to their target psychiatric phenotypes. Family and twin designs capture a broader component of inherited and familial variation than molecular PRS and could therefore yield larger associations with environmental measures, although such estimates are not directly comparable and may also incorporate shared familial influences. UK Biobank selection, the middle-to-older age range, European-ancestry restriction and predominantly European-ancestry discovery GWAS limit generalisability.

## Conclusion

Polygenic liability was associated with a broad but patterned set of environmental, behavioural and subjective risk factors. Schizophrenia liability was related particularly to smoking, cannabis use, deprivation, interpersonal adversity and the experienced quality of close relationships, while other scores showed distinguishable profiles. The strongest conceptual finding was that psychiatric liability was more closely associated with perception/subjectivity than with objective/non-perception factors, including in matched social comparisons. Psychiatric exposome research should therefore consider external exposure and subjective experience separately while recognising that both may reflect interactions among inherited liability, social circumstances, behavioural pathways and cognitive appraisal.

## Methods

### Participants and study design

UK Biobank is a population-based prospective cohort of approximately 500,000 participants recruited across the United Kingdom between 2006 and 2010^42,43^. The present analysis included 307,493 participants aged 38-73 years with eligible genotype and phenotype data; 52% were female and 48% male.

Participants with an ICD-10 diagnosis of schizophrenia, bipolar disorder, or major depressive disorder, or recorded antipsychotic medication use, were excluded to reduce the potential influence of diagnosed psychiatric disorder or its treatment on the environmental risk-factor measures. Each PRS-environmental risk-factor association included participants with available data for the relevant environmental risk factor, PRS, age, sex, the first 15 genetic principal components, genotyping batch, and assessment centre. Analytic sample sizes ranged from 43,333 to 304,634 across the 441 models.

All participants provided written informed consent. UK Biobank received ethical approval from the North West Multi-centre Research Ethics Committee (REC reference 11/NW/0382). The study was conducted under UK Biobank application 18177 and preregistered on the Open Science Framework on 16 August 2019 (osf.io/4au38).

### Genotype quality control, ancestry and relatedness

Blood samples from 488,366 UK Biobank participants were genotyped using the UK BiLEVE array or UK Biobank Axiom array^43^. Variants were excluded for genotype missingness greater than 0.02, minor allele frequency below 0.01, or Hardy-Weinberg equilibrium *P* < 1 × 10⁻⁸. Participants were excluded for genotype missingness greater than 0.01, discordance between reported and genetically inferred sex, or outlying heterozygosity and missingness according to UK Biobank recommendations.

Participants of inferred European genetic ancestry were identified using four-means clustering of the first two genetic principal components. One participant from each related pair was excluded using a KING kinship coefficient threshold of 0.088. The first 15 genetic principal components were included as covariates in all association models.

### GWAS data and polygenic-score generation

Nine PRS represented liabilities relevant to psychosis, affective psychopathology, neurodevelopment, personality, cognition and socioeconomic position, together with non-psychiatric comparison traits. Summary statistics were obtained for schizophrenia, bipolar disorder, major depression, ADHD, neuroticism, extraversion, educational attainment, Alzheimer’s disease and LDL cholesterol^33–37,44–47^. The discovery summary statistics used in the final analysis did not include UK Biobank participants. Discovery-GWAS characteristics and score-construction details are reported in Supplementary Table 2.

PRS were generated using PRS-CS, which estimates posterior SNP effect sizes using continuous-shrinkage priors and an external linkage-disequilibrium reference panel^48^. GWAS summary statistics were harmonised to HapMap3 variants and processed chromosome by chromosome using the European 1000 Genomes linkage-disequilibrium reference panel. Posterior effects were scored in UK Biobank genotype data in PLINK binary format after allele alignment; incompatible alleles and unresolved strand-ambiguous variants were excluded. Each PRS was standardised to mean zero and standard deviation one before analysis.

### Environmental risk factors and final categories

Forty-nine environmental risk factors were selected from the epidemiological literature on environmental risk for schizophrenia and related psychiatric outcomes^4–11,49^. Binary, ordinal and continuous measures were retained on their available coded scales and recoded where necessary so that higher values represented greater exposure, adversity, poorer circumstances or the hypothesised higher-risk direction. Each measure was standardised before regression. UK Biobank fields, question wording, analytic coding, availability and descriptive statistics are reported in Supplementary Table 1.

The 49 measures were assigned to five conceptually defined categories: (1) perception/subjectivity (7 measures), covering perceived health, finances, relationships and social disconnection; (2) interpersonal adversity/victimisation (6 measures), covering childhood maltreatment, assault, violent crime and partner violence; (3) objective-life events (9 measures); (4) objective-socioeconomic/environmental factors (16 measures); and (5) behavioural/lifestyle factors (11 measures). The categories were based on item content and measurement process, were not empirically derived, and were not interpreted as single latent constructs.

### Individual association analyses and multiple testing

Each of the nine standardised PRS was tested separately against each of the 49 standardised environmental risk factors, producing 441 models. The environmental risk factor was the outcome and the PRS the predictor. Models were adjusted for age, sex, the first 15 genetic principal components, genotyping batch and assessment centre and estimated using linear regression with HC1 heteroskedasticity-consistent robust standard errors. Genotyping batch and assessment centre were entered as categorical factors. The coefficient represents the standard-deviation difference in the environmental risk factor associated with a one-standard-deviation increase in PRS.

Missing data were handled separately for each environmental risk factor. The Benjamini-Hochberg false-discovery-rate procedure was applied across the 49 tests separately for each PRS; FDR correction across all 441 models was additionally calculated for supplementary reporting. Full model estimates and model-specific sample sizes are reported in Supplementary Table 3.

### Category-level analyses

For each PRS, estimates were combined within categories using inverse-variance fixed-effect pooling: *β̂* = Σ(*w*ᵢ*β*ᵢ)/Σwᵢ, where *w*ᵢ = 1/SEᵢ². The pooled standard error was SE(*β̂*) = √[1/Σwᵢ]. Differences between two pooled estimates were tested using *z* = (*β̂*₁ − *β̂*₂)/√[SE(*β̂*₁)² + SE(*β̂*₂)²], with two-sided P-values from the standard normal distribution.

Because measures within each category were heterogeneous and correlated, pooled estimates were treated as descriptive summaries of direction and relative magnitude rather than effects of single underlying constructs. Category estimates and contrasts are reported in Supplementary Table 4. Model-specific sample size varied across ERFs, so precision and statistical power also varied across associations; inverse-variance pooling therefore gave greater weight to more precisely estimated effects. Differences in phenotype reliability may additionally attenuate standardised associations and should be considered when comparing ERFs.

### Perception/subjectivity and objective/non-perception analyses

The broad comparison pooled the seven perception/subjectivity measures and contrasted them with 25 objective/non-perception measures comprising the objective-life-event and objective-socioeconomic/environmental categories. All six interpersonal adversity/victimisation measures and all 11 behavioural/lifestyle measures were excluded. This was to prevent assault, abuse and victimisation from being characterised as perception-based outcomes and to avoid forcing behavioural measures into either side of the comparison.

The primary matched analysis compared four conceptually related pairs: loneliness versus behavioural social isolation; happiness with own health versus overall health rating; financial situation satisfaction versus ability to pay rent or mortgage; and family relationship satisfaction versus frequency of friend or family visits. The comparator in each pair was considered comparatively less dependent on perception, not wholly independent of self-report or measurement error.

Broad and matched estimates were pooled using the same inverse-variance procedure and compared using the corresponding Wald statistic. For the matched analyses, Benjamini-Hochberg FDR correction was additionally applied across the 36 individual pair-specific contrast tests and across the nine pooled four-pair contrasts. Full results are reported in Supplementary Table 5.

## Supplementary information

Supplementary Information

## Data Availability

UK Biobank data are available to approved researchers through UK Biobank access procedures. GWAS summary statistics used for PRS construction are available from the contributing consortia or public repositories under their respective terms. Software: Data preparation and statistical analyses were conducted in R version 4.5.2 (data.table version 1.14.6); figures were generated using ggplot2 version 4.0.3 and patchwork version 1.3.2. HC1 heteroskedasticity-consistent standard errors were calculated directly from the model matrix and residuals using the finite-sample correction n/(n-p). PRS-CS was run in Python version 3.11.5 using repository commit 53303906784c3360b9a1a7fa000b32c73eda9d5a (21 November 2024), and genotype scoring was performed using PLINK.

## Code availability

Analysis and figure-generation scripts will be made available with the final article and are included in the accompanying reproducibility package.
