# Supplementary material for: Genetic risk of psychopathology predicts environmental risk
Source: Schizophrenia (Heidelb). 2026 Oct 6;12(1):85. doi: 10.1038/s41537-026-00805-3 (PMC13743761; doi:10.1038/s41537-026-00805-3)
Supplement: Supplementary file 1 [file 41537_2026_805_MOESM1_ESM.docx]

**Supplementary Information
Genetic risk of psychopathology predicts environmental risk**

# Supplementary Table 1. Environmental risk-factor definitions, coding and final classification

| **No.** | **UKB-derived variable** | **Biobank question/measure** | **Original coding** | **Manuscript risk label** | **Final ERF category** | **Broad comparison class** | **Analytic N** | **Missing N** | **Current analytic scale/range** | **Mean** | **SD** |
| --- | --- | --- | --- | --- | --- | --- | --- | --- | --- | --- | --- |
| 1 | Smoking.status.0.0 | Do you smoke tobacco now? | 0=never 1=previous 2=current | Smoking | Behavioural/lifestyle | Excluded: behavioural/lifestyle | 303330 | 1304 | 0, 1, 2 | 0.521730042555415 | 0.644074754854644 |
| 2 | Alcohol.intake.frequency.0.0 | About how often do you drink alcohol? | 6=Daily or almost daily 5=Three or four times a week 4=Once or twice a week 3=One to three times a month 2=Special occasions only 1=Never | Frequent alcohol intake | Behavioural/lifestyle | Excluded: behavioural/lifestyle | 304278 | 356 | 1, 2, 3, 4, 5, 6 | 4.19449952301416 | 1.45203916221616 |
| 3 | Number.of.days.week.of.moderate.physical.activity.10.minutes.0.0 | In a typical WEEK, on how many days did you do 10 minutes or more of moderate physical activities like carrying light loads, cycling at normal pace? (Do not include walking) | 0-7 days 0=7, 7=0 | Low moderate physical activity | Behavioural/lifestyle | Excluded: behavioural/lifestyle | 290819 | 13940 | 0, 1, 2, 3, 4, 5, 6, 7 | 3.66579799899848 | 2.31559556317799 |
| 4 | Number.of.days.week.of.vigorous.physical.activity.10.minutes.0.0 | In a typical WEEK, how many days did you do 10 minutes or more of vigorous physical activity? (These are activities that make you sweat or breathe hard such as fast cycling, aerobics, heavy lifting) | 0-7 days 0=7, 7=0 | Low vigorous physical activity | Behavioural/lifestyle | Excluded: behavioural/lifestyle | 290787 | 13976 | 0, 1, 2, 3, 4, 5, 6, 7 | 1.88316519997138 | 1.95382061879648 |
| 5 | Vegetable.consumers.0.0 | Did you eat any beans, lentils, potatoes or vegetables yesterday? Include fresh, tinned, frozen, dried; chips, salad, coleslaw, baked beans, chickpeas, veg in stews, pies etc. | 0=yes 1=no | Low vegetable consumption | Behavioural/lifestyle | Excluded: behavioural/lifestyle | 43333 | 263762 | 0, 1 | 0.173446754018888 | 0.37863736728688 |
| 6 | Fish.consumer.0.0 | Did you eat any fish or seafood yesterday? e.g. at breakfast, takeaway with chips, smoked fish, fish pate, tuna in sandwiches. | 0=yes 1=no | Low fish consumption | Behavioural/lifestyle | Excluded: behavioural/lifestyle | 43333 | 263762 | 0, 1 | 0.70711852004299 | 0.455089721459775 |
| 7 | Fruit.consumers.0.0 | Did you eat any fruit yesterday? | 0=yes 1=no | Low fruit consumption | Behavioural/lifestyle | Excluded: behavioural/lifestyle | 43333 | 263762 | 0, 1 | 0.179597996844344 | 0.38385664738804 |
| 8 | Yogurt.ice.cream.consumers.0.0 | Did you eat any yogurt or ice-cream yesterday? | 0=yes 1=no | Low yogurt/ice cream consumption | Behavioural/lifestyle | Excluded: behavioural/lifestyle | 43333 | 263762 | 0, 1 | 0.597059294322106 | 0.490494643049752 |
| 9 | Ever.taken.cannabis | Have you taken CANNABIS (marijuana, grass, hash, ganja, blow, draw, skunk, weed, spliff, dope), even if it was a long time ago? | 0=No 1=Yes, 1-2 times 2=Yes, 3-10 times 3=Yes, 11-100 times 4=Yes, more than 100 times | Ever taken cannabis | Behavioural/lifestyle | Excluded: behavioural/lifestyle | 83055 | 223764 | 0, 1, 2, 3, 4 | 0.40942803568656 | 0.926249198507288 |
| 10 | Marital.separation.divorce | In the last 2 years have you experienced any of the following? (You can select more than one answer) | 0=no 1=yes | Marital separation or divorce | Objective life events | Objective/non-perception | 304634 | 0 | 0, 1 | 0.0268233748410533 | 0.161567219125878 |
| 11 | Sexual.interference.by.partner.or.ex.partner.without.consent.as.an.adult | Since I was sixteen... A partner or ex-partner sexually interfered with me, or forced me to have sex against my wishes | 0=Never true 1=Rarely true 2=Sometimes true 3=Often 4=Very often true | Sexual interference by partner/ex-partner without consent | Interpersonal adversity/victimisation | Excluded: interpersonal adversity/victimisation | 82967 | 223853 | 0, 1, 2, 3, 4 | 0.0572931611669058 | 0.321245825757069 |
| 12 | Victim.of.sexual.assault | In your life, have you...? Been a victim of a sexual assault, whether by a stranger or someone you knew | 0=Never 1=Yes, but not in the last 12 months 2=Yes, within the last 12 months | Victim of sexual assault | Interpersonal adversity/victimisation | Excluded: interpersonal adversity/victimisation | 82369 | 224453 | 0, 1, 2 | 0.116979768786127 | 0.323638456503865 |
| 13 | Belittlement.by.partner.or.ex.partner.as.an.adult | Since I was sixteen... A partner or ex-partner repeatedly belittled me to the extent that I felt worthless | 0=Never true 1=Rarely true 2=Sometimes true 3=Often 4=Very often true | Belittlement by partner/ex-partner | Perception/subjectivity | Perception/subjectivity | 82956 | 223864 | 0, 1, 2, 3, 4 | 0.3167561491827 | 0.752470219793049 |
| 14 | Physical.violence.by.partner.or.ex.partner.as.an.adult | Since I was sixteen... A partner or ex-partner deliberately hit me or used violence in any other way | 0=Never true 1=Rarely true 2=Sometimes true 3=Often 4=Very often true | Physical violence by partner/ex-partner | Interpersonal adversity/victimisation | Excluded: interpersonal adversity/victimisation | 82976 | 223844 | 0, 1, 2, 3, 4 | 0.153570275795287 | 0.529260911676198 |
| 15 | Victim.of.physically.violent.crime | "In your life, have you...? Been attacked, mugged, robbed, or been the victim of a physically violent crime" | 0=Never 1=Yes, but not in the last 12 months 2=Yes, within the last 12 months | Victim of physically violent crime | Interpersonal adversity/victimisation | Excluded: interpersonal adversity/victimisation | 83062 | 223758 | 0, 1, 2 | 0.184259867438944 | 0.39794379342844 |
| 16 | Felt.hated.by.family.member.as.a.child | When I was growing up... I felt that someone in my family hated me | 0=Never true 1=Rarely true 2=Sometimes true 3=Often 4=Very often true | Felt hated by family member as a child | Perception/subjectivity | Perception/subjectivity | 83007 | 223814 | 0, 1, 2, 3, 4 | 0.196907228814876 | 0.614236321884402 |
| 17 | Physically.abused.by.family.as.a.child | When I was growing up... People in my family hit me so hard that it left me with bruises or marks | 0=Never true 1=Rarely true 2=Sometimes true 3=Often 4=Very often true | Physically abused by family as a child | Interpersonal adversity/victimisation | Excluded: interpersonal adversity/victimisation | 83033 | 223786 | 0, 1, 2, 3, 4 | 0.232608981327726 | 0.588213529219936 |
| 18 | Sexually.molested.as.a.child | When I was growing up... Someone molested me (sexually) | 0=Never true 1=Rarely true 2=Sometimes true 3=Often 4=Very often true | Sexually molested as a child | Interpersonal adversity/victimisation | Excluded: interpersonal adversity/victimisation | 82479 | 224343 | 0, 1, 2, 3, 4 | 0.102285027059531 | 0.423416296932165 |
| 19 | Family.relationship.satisfaction.0.0 | In general how satisfied are you with your FAMILY RELATIONSHIPS? | 1=Extremely happy 2=Very happy 3=Moderately happy 4=Moderately unhappy 5=Very unhappy 6=Extremely unhappy | Low family relationship satisfaction | Perception/subjectivity | Perception/subjectivity | 99904 | 206652 | 1, 2, 3, 4, 5, 6 | 2.1785186580855 | 0.866668169461303 |
| 20 | Been.involved.in.combat.or.exposed.to.war.zone | In your life, have you...? Been involved in combat or exposed to a war-zone (either in the military or as a civilian) | 0=Never 1=Yes, but not in the last 12 months 2=Yes, within the last 12 months | Combat or war-zone exposure | Objective life events | Objective/non-perception | 83097 | 223724 | 0, 1, 2 | 0.0379257243132901 | 0.194792717330159 |
| 21 | Financial.difficulties | In the last 2 years have you experienced any of the following? (You can select more than one answer) | 0=no 1=yes | Financial difficulties | Objective socioeconomic/environmental | Objective/non-perception | 304634 | 0 | 0, 1 | 0.0983079289609845 | 0.29773103355775 |
| 22 | Financial.situation.satisfaction.0.0 | In general how satisfied are you with your FINANCIAL SITUATION? | 1=Extremely happy 2=Very happy 3=Moderately happy 4=Moderately unhappy 5=Very unhappy 6=Extremely unhappy | Low financial situation satisfaction | Perception/subjectivity | Perception/subjectivity | 100315 | 206231 | 1, 2, 3, 4, 5, 6 | 2.65785783413324 | 0.9092938383584 |
| 23 | In.paid.employment.or.self.employed | Which of the following describes your current situation? (You can select more than one answer) | 0=yes 1=no | Not in paid employment/self-employment | Objective socioeconomic/environmental | Objective/non-perception | 304634 | 0 | 0, 1 | 0.414559030612079 | 0.492646556913129 |
| 24 | Unable.to.work.because.of.sickness.or.disability | Which of the following describes your current situation? (You can select more than one answer) | 0=no 1=yes | Unable to work because of sickness/disability | Objective socioeconomic/environmental | Objective/non-perception | 304634 | 0 | 0, 1 | 0.0215224411612622 | 0.145118207592262 |
| 25 | Unemployed | Which of the following describes your current situation? (You can select more than one answer) | 0=no 1=yes | Unemployed | Objective socioeconomic/environmental | Objective/non-perception | 304634 | 0 | 0, 1 | 0.0149824548851519 | 0.121482628080927 |
| 26 | Able.to.pay.rent.mortgage.as.an.adult | Since I was sixteen... There was money to pay the rent or mortgage when I needed it | 5=Never true 4=Rarely true 3=Sometimes true 2=Often 1=Very often true | Unable to pay rent/mortgage as an adult | Objective socioeconomic/environmental | Objective/non-perception | 81997 | 224830 | 1, 2, 3, 4, 5 | 1.2427083459347 | 0.79709027878245 |
| 27 | Own.or.rent.accommodation.lived.in.0.0 | Do you own or rent the accommodation that you live in | 1 = Own outright (by you or someone in your household) 2 = Own with a mortgage 3 = Rent - from local authority, local council, housing association 4 = Rent - from private landlord or letting agency 5 = Pay part rent and part mortgage (shared ownership) 6 = Live in accommodation rent free | Non-owner accommodation / renting | Objective socioeconomic/environmental | Objective/non-perception | 300624 | 4046 | 1, 2, 3, 4, 5, 6 | 1.57618628623779 | 0.803003540739677 |
| 28 | Average.total.household.income.before.tax.0.0 | What is the average total income before tax received by your HOUSEHOLD | 5= Less than 18,000 4= 18,000 to 30,999 3= 31,000 to 51,999 2= 52,000 to 100,000 1= Greater than 100,000 | Low average household income | Objective socioeconomic/environmental | Objective/non-perception | 261733 | 43273 | 1, 2, 3, 4, 5 | 3.30968132616759 | 1.19008286265566 |
| 29 | Particulate.matter.air.pollution.pm10.2010.0.0 | PM10 (particulate matter with diameter less than or equal to 10 micrometres); Land Use Regression (LUR) estimate for annual average 2010. | 11.78-31.39 micro-g/m3 | Higher PM10 air pollution | Objective socioeconomic/environmental | Objective/non-perception | 277926 | 26957 | -2.303 to 7.987 | -8.92383710215443e-06 | 0.99999479882943 |
| 30 | Particulate.matter.air.pollution.pm2.5.2010.0.0 | Particulate matter air pollution (PM2.5); residential Land Use Regression estimate for annual average 2010 (UK Biobank Data-Field 24006). | Continuous, micrograms/m3; higher values indicate greater PM2.5 exposure. | Higher PM2.5 air pollution | Objective socioeconomic/environmental | Objective/non-perception | 277926 | 26957 | -1.687 to 10.922 | -1.81008149292751e-05 | 0.999992936207563 |
| 31 | Maternal.smoking.around.birth.0.0 | Did your mother smoke regularly around the time when you were born? | 0=no 1=yes | Maternal smoking around birth | Behavioural/lifestyle | Excluded: behavioural/lifestyle | 261771 | 43299 | 0, 1 | 0.296645646759578 | 0.456779812130949 |
| 32 | Nitrogen.dioxide.air.pollution.2010.0.0 | Nitrogen dioxide; Land Use Regression (LUR) estimate for annual average 2010. | 12.93-108.49 micro-g/m3 | Higher nitrogen dioxide air pollution | Objective socioeconomic/environmental | Objective/non-perception | 300348 | 4341 | -1.784 to 11.078 | -3.51264452897056e-06 | 1.00001038562268 |
| 33 | Townsend.deprivation.index.at.recruitment.0.0 | Townsend deprivation index calculated immediately prior to participant joining UK Biobank. Based on the preceding national census output areas. Each participant is assigned a score corresponding to the output area in which their postcode is located. | -6.25826-11.0013 | Higher Townsend deprivation | Objective socioeconomic/environmental | Objective/non-perception | 304310 | 328 | -1.601 to 4.315 | 4.89844239147956e-06 | 1.00000730370057 |
| 34 | Home.area.population.density.urban.or.rural.0.0 | The classification is derived by combining each participants home postcode with data generated from the 2001 census from the Office of National Statistics, using the Geoconvert tool from Census Dissemination Unit. | 7=England/Wales - Urban - sparse 5=England/Wales - Town and Fringe - sparse 3=England/Wales - Village - sparse 1=England/Wales - Hamlet and Isolated dwelling - sparse 8=England/Wales - Urban - less sparse 6=England/Wales - Town and Fringe - less sparse 4=England/Wales - Village - less sparse 2=England/Wales - Hamlet and Isolated Dwelling - less sparse 8=Scotland - Large Urban Area 7=Scotland - Other Urban Area 6=Scotland - Accessible Small Town 5=Scotland - Remote Small Town 4=Scotland - Very Remote Small Town 3=Scotland - Accessible Rural 2=Scotland - Remote Rural 1=Scotland - Very Remote Rural | Urban/rural population density | Objective socioeconomic/environmental | Objective/non-perception | 301809 | 2855 | 1, 2, 3, 4, 5, 6, 7, 8 | 2.03071842646026 | 2.13701208052517 |
| 35 | General.happiness.with.own.health | In general how happy are you with your HEALTH? | 1=Extremely happy 2=Very happy 3=Moderately happy 4=Moderately unhappy 5=Very unhappy 6=Extremely unhappy | Low happiness with own health | Perception/subjectivity | Perception/subjectivity | 83016 | 223804 | 1, 2, 3, 4, 5, 6 | 2.49311140054249 | 0.851963708402033 |
| 36 | Overall.health.rating.0.0 | In general how would you rate your overall health? | 1 Excellent 2 Good 3 Fair 4 Poor | Poor overall health rating | Perception/subjectivity | Perception/subjectivity | 303427 | 1222 | 1, 2, 3, 4 | 2.05275719869005 | 0.690573287672308 |
| 37 | Been.in.serious.accident.believed.to.be.life.threatening | In your life, have you...? Been in a serious accident that you believed to be life-threatening at the time | 0=Never 1=Yes, but not in the last 12 months 2=Yes, within the last 12 months | Serious life-threatening accident | Objective life events | Objective/non-perception | 83098 | 223721 | 0, 1, 2 | 0.0925130115074249 | 0.298596753995933 |
| 38 | Diagnosed.with.life.threatening.illness | In your life, have you...? Been diagnosed with a life-threatening illness | 0=Never 1=Yes, but not in the last 12 months 2=Yes, within the last 12 months | Diagnosed with life-threatening illness | Objective life events | Objective/non-perception | 82917 | 223908 | 0, 1, 2 | 0.17138242507627 | 0.426216992935275 |
| 39 | Serious.illness.injury.or.assault.to.yourself | In the last 2 years have you experienced any of the following? (You can select more than one answer) | Own health | Serious illness/injury/assault to self | Objective life events | Objective/non-perception | 304634 | 0 | 0, 1 | 0.080827856243882 | 0.272571010039607 |
| 40 | Health.sumscore | Has a doctor ever told you that you have had any of the following conditions? (You can select more than one answer) | Score between 0 and 12 comprising diagnoses by doctor of:  Diabetes Cancer Other serious medical condition/disability Heart attack Angina Stroke High blood pressure DVT Blood clot lung Emphysema Asthma Allergies | Poor health sum score | Objective socioeconomic/environmental | Objective/non-perception | 304634 | 0 | 0, 1, 2, 3, 4, 5, 6, 7, 8, 9 | 0.767588205259957 | 0.9438975048204 |
| 41 | Father.still.alive.0.0 | Is your father still alive? | Is your father still alive? | Father not alive | Objective socioeconomic/environmental | Objective/non-perception | 296617 | 8106 | 0, 1 | 0.767962536783494 | 0.422133478991741 |
| 42 | Mother.still.alive.0.0 | Is your mother still alive? | 0=yes 1=no | Mother not alive | Objective socioeconomic/environmental | Objective/non-perception | 299550 | 5137 | 0, 1 | 0.60178068237442 | 0.489531904247261 |
| 43 | Non.accidental.death.in.close.genetic.family.0.0 | Have any of your mother, father, brothers or sisters died suddenly from a non-accidental cause? (Do not include half-, step- or adopted brothers and sisters) | 0=no 1=yes | Non-accidental death in close genetic family | Objective life events | Objective/non-perception | 95591 | 211009 | 0, 1 | 0.268396832635463 | 0.443127530225345 |
| 44 | Death.of.a.close.relative | In the last 2 years have you experienced any of the following? (You can select more than one answer) | 0=no 1=yes | Death of a close relative | Objective life events | Objective/non-perception | 304634 | 0 | 0, 1 | 0.204583518974416 | 0.40339761024647 |
| 45 | Death.of.a.spouse.or.partner | In the last 2 years have you experienced any of the following? (You can select more than one answer) | 0=no 1=yes | Death of spouse or partner | Objective life events | Objective/non-perception | 304634 | 0 | 0, 1 | 0.0143710588533723 | 0.119015030922656 |
| 46 | Serious.illness.injury.or.assault.of.a.close.relative | In the last 2 years have you experienced any of the following? (You can select more than one answer) | 0=no 1=yes | Serious illness/injury/assault of close relative | Objective life events | Objective/non-perception | 304634 | 0 | 0, 1 | 0.110334218990351 | 0.313306396902203 |
| 47 | Frequency.of.friend.family.visits.0.0 | How often do you visit friends or family or have them visit you? | 1= Almost daily 2= 2-4 times a week 3= About once a week 4= About once a month 5= Once every few months 6= Never or almost never 7= No friends/family outside household | Infrequent friend/family visits | Objective socioeconomic/environmental | Objective/non-perception | 302589 | 2059 | 1, 2, 3, 4, 5, 6, 7 | 2.77448483142021 | 1.11654429786218 |
| 48 | Loneliness | UK Biobank touchscreen item: "Do you often feel lonely?" (Data-Field 2020: Loneliness, isolation). | No = 0; Yes = 1. Higher values indicate loneliness. | Loneliness/isolation | Perception/subjectivity | Perception/subjectivity | 300002 | 4670 | 0, 1 | 0.14812283082857 | 0.35522228886212 |
| 49 | Social.isolation.behaviour | Derived behavioural social-isolation score combining four UK Biobank indicators of socially isolating behaviour, as defined in Socrates et al. (2024). | Derived score ranging from 1 to 18; higher values indicate greater behavioural social isolation. | Social isolation behaviour | Behavioural/lifestyle | Excluded: behavioural/lifestyle | 304634 | 0 | 1 to 18 | 8.24370636079521 | 2.56068308310294 |

# Supplementary Table 2. Discovery GWAS and PRS construction

| **PRS** | **Discovery GWAS / exact source** | **PRS-CS input** | **Variant count (see note)** |
| --- | --- | --- | --- |
| Schizophrenia | Trubetskoy et al. (2022); PGC3_SCZ_wave3.european.autosome.public.v3.vcf.tsv.gz; European autosomes | n_gwas=58,749; 527,931 unique input variants | 150,859 posterior |
| Bipolar disorder | PGC 2024 European analysis excluding UK Biobank and 23andMe; bip2024_eur_noUKB_no23andMe.gz | n_gwas=75,764; 562,501 unique input variants | 151,378 posterior |
| Major depression | PGC 2025 European analysis excluding UK Biobank and 23andMe; pgc-mdd2025_no23andMe-noUKBB_eur_v3-49-24-11_formatted.tsv.gz | n_gwas=956,987; 596,675 unique input variants | 145,405 posterior |
| ADHD | Demontis et al. (2023); ADHD2022_iPSYCH_deCODE_PGC.meta.gz | n_gwas=127,902; 487,809 unique input variants | 140,108 posterior |
| Educational attainment | Okbay et al. (2016); SSGAC discovery GWAS (UK Biobank used only for independent replication and not included in the discovery summary statistics used here) | n_gwas=293,723; 1,103,277 unique input variants | 149,485 scored |
| Neuroticism | De Moor et al. (2015); Neuro_GPC2 source | n_gwas=63,661; 476,018 unique input variants | 143,884 scored |
| Extraversion | van den Berg et al. (2016); Extra_GPC2 source | n_gwas=63,030; 475,742 unique input variants | 143,802 scored |
| Alzheimer’s disease | Kunkle et al. (2019); Stage 1 clinically diagnosed Alzheimer’s disease GWAS (21,982 cases; 41,944 controls) | n_gwas=63,926; 1,117,490 unique input variants | 153,727 scored |
| LDL cholesterol | Graham et al. (2021); European analysis excluding UK Biobank; without_UKB_LDL_INV_EUR_HRC_1KGP3_others_ALL.meta.singlevar.results.gz | n_gwas=836,259; 722,670 unique input variants | 155,313 posterior |

All scores were generated with PRS-CS using the European 1000 Genomes LD reference, HapMap3-aligned inputs and phi estimated automatically. PRS-CS was run in Python; genotype scoring was performed using PLINK. None of the discovery summary statistics used for the final PRS included UK Biobank participants. Educational attainment used the 293,723-participant Okbay et al. discovery sample; UK Biobank was used only as an independent replication sample in that publication and was not part of the discovery summary statistics used here. Variant counts for the five unchanged PRS are retained posterior-variant counts from the existing score records; for the four repaired PRS (educational attainment, Alzheimer’s disease, neuroticism and extraversion), counts are the variants scored in the final target genotype data.

# Supplementary Table 3. All 441 PRS–environmental risk-factor associations

| **PRS** | **Environmental risk factor** | **n** | **beta** | **se** | **p** | **fdr_within_prs** | **fdr_all** | **Final ERF category** |
| --- | --- | --- | --- | --- | --- | --- | --- | --- |
| Alzheimer's disease | Smoking | 303330 | -0.0022 | 0.0018 | 0.2068 | 0.6756 | 0.3293 | Behavioural/lifestyle |
| Alzheimer's disease | Frequent alcohol intake | 304278 | 0.0009 | 0.0018 | 0.6242 | 0.8081 | 0.7522 | Behavioural/lifestyle |
| Alzheimer's disease | Low moderate physical activity | 290819 | 0.0099 | 0.0018 | 8.11e-8 | 9.93e-7 | 3.06e-7 | Behavioural/lifestyle |
| Alzheimer's disease | Low vigorous physical activity | 290787 | 0.0104 | 0.0018 | 1.90e-8 | 4.66e-7 | 7.76e-8 | Behavioural/lifestyle |
| Alzheimer's disease | Low vegetable consumption | 43333 | 0.0006 | 0.0048 | 0.9076 | 0.9696 | 0.9530 | Behavioural/lifestyle |
| Alzheimer's disease | Low fish consumption | 43333 | 0.0020 | 0.0048 | 0.6789 | 0.8318 | 0.7938 | Behavioural/lifestyle |
| Alzheimer's disease | Low fruit consumption | 43333 | -0.0026 | 0.0047 | 0.5783 | 0.8081 | 0.7164 | Behavioural/lifestyle |
| Alzheimer's disease | Low yogurt/ice cream consumption | 43333 | -0.0015 | 0.0048 | 0.7486 | 0.8337 | 0.8422 | Behavioural/lifestyle |
| Alzheimer's disease | Ever taken cannabis | 83055 | -0.0003 | 0.0033 | 0.9389 | 0.9789 | 0.9689 | Behavioural/lifestyle |
| Alzheimer's disease | Marital separation or divorce | 304634 | 3.84e-5 | 0.0018 | 0.9831 | 0.9850 | 0.9939 | Objective life events |
| Alzheimer's disease | Sexual interference by partner/ex-partner without consent | 82967 | -0.0052 | 0.0034 | 0.1287 | 0.4852 | 0.2217 | Interpersonal adversity/victimisation |
| Alzheimer's disease | Victim of sexual assault | 82369 | 0.0037 | 0.0035 | 0.2878 | 0.7676 | 0.4217 | Interpersonal adversity/victimisation |
| Alzheimer's disease | Belittlement by partner/ex-partner | 82956 | 0.0064 | 0.0034 | 0.0628 | 0.2797 | 0.1183 | Perception/subjectivity |
| Alzheimer's disease | Physical violence by partner/ex-partner | 82976 | 0.0013 | 0.0034 | 0.7043 | 0.8318 | 0.8122 | Interpersonal adversity/victimisation |
| Alzheimer's disease | Victim of physically violent crime | 83062 | -0.0058 | 0.0034 | 0.0887 | 0.3621 | 0.1623 | Interpersonal adversity/victimisation |
| Alzheimer's disease | Felt hated by family member as a child | 83007 | 0.0011 | 0.0034 | 0.7407 | 0.8337 | 0.8376 | Perception/subjectivity |
| Alzheimer's disease | Physically abused by family as a child | 83033 | -0.0026 | 0.0035 | 0.4595 | 0.8081 | 0.6031 | Interpersonal adversity/victimisation |
| Alzheimer's disease | Sexually molested as a child | 82479 | 0.0019 | 0.0035 | 0.5907 | 0.8081 | 0.7276 | Interpersonal adversity/victimisation |
| Alzheimer's disease | Low family relationship satisfaction | 99904 | 0.0034 | 0.0031 | 0.2794 | 0.7676 | 0.4116 | Perception/subjectivity |
| Alzheimer's disease | Combat or war-zone exposure | 83097 | -0.0028 | 0.0034 | 0.4054 | 0.8081 | 0.5435 | Objective life events |
| Alzheimer's disease | Financial difficulties | 304634 | -0.0013 | 0.0018 | 0.4816 | 0.8081 | 0.6247 | Objective socioeconomic/environmental |
| Alzheimer's disease | Low financial situation satisfaction | 100315 | 0.0018 | 0.0031 | 0.5713 | 0.8081 | 0.7154 | Perception/subjectivity |
| Alzheimer's disease | Not in paid employment/self-employment | 304634 | -0.0005 | 0.0015 | 0.7130 | 0.8318 | 0.8124 | Objective socioeconomic/environmental |
| Alzheimer's disease | Unable to work because of sickness/disability | 304634 | 0.0015 | 0.0018 | 0.3961 | 0.8081 | 0.5341 | Objective socioeconomic/environmental |
| Alzheimer's disease | Unemployed | 304634 | -0.0009 | 0.0018 | 0.6267 | 0.8081 | 0.7530 | Objective socioeconomic/environmental |
| Alzheimer's disease | Unable to pay rent/mortgage as an adult | 81997 | -0.0048 | 0.0034 | 0.1604 | 0.5613 | 0.2669 | Objective socioeconomic/environmental |
| Alzheimer's disease | Non-owner accommodation / renting | 300624 | -0.0008 | 0.0017 | 0.6228 | 0.8081 | 0.7522 | Objective socioeconomic/environmental |
| Alzheimer's disease | Low average household income | 261733 | 0.0011 | 0.0018 | 0.5406 | 0.8081 | 0.6831 | Objective socioeconomic/environmental |
| Alzheimer's disease | Higher PM10 air pollution | 277926 | -0.0021 | 0.0018 | 0.2448 | 0.7499 | 0.3711 | Objective socioeconomic/environmental |
| Alzheimer's disease | Higher PM2.5 air pollution | 277926 | -0.0057 | 0.0017 | 0.0010 | 0.0102 | 0.0026 | Objective socioeconomic/environmental |
| Alzheimer's disease | Maternal smoking around birth | 261771 | -0.0036 | 0.0019 | 0.0608 | 0.2797 | 0.1151 | Behavioural/lifestyle |
| Alzheimer's disease | Higher nitrogen dioxide air pollution | 300348 | -0.0045 | 0.0016 | 0.0041 | 0.0287 | 0.0095 | Objective socioeconomic/environmental |
| Alzheimer's disease | Higher Townsend deprivation | 304310 | -0.0054 | 0.0017 | 0.0013 | 0.0102 | 0.0031 | Objective socioeconomic/environmental |
| Alzheimer's disease | Urban/rural population density | 301809 | -2.23e-5 | 0.0012 | 0.9850 | 0.9850 | 0.9939 | Objective socioeconomic/environmental |
| Alzheimer's disease | Low happiness with own health | 83016 | 0.0022 | 0.0035 | 0.5275 | 0.8081 | 0.6685 | Perception/subjectivity |
| Alzheimer's disease | Poor overall health rating | 303427 | -0.0014 | 0.0018 | 0.4348 | 0.8081 | 0.5776 | Perception/subjectivity |
| Alzheimer's disease | Serious life-threatening accident | 83098 | -0.0086 | 0.0034 | 0.0121 | 0.0742 | 0.0259 | Objective life events |
| Alzheimer's disease | Diagnosed with life-threatening illness | 82917 | 0.0025 | 0.0034 | 0.4690 | 0.8081 | 0.6119 | Objective life events |
| Alzheimer's disease | Serious illness/injury/assault to self | 304634 | 0.0002 | 0.0018 | 0.9102 | 0.9696 | 0.9530 | Objective life events |
| Alzheimer's disease | Poor health sum score | 304634 | -0.0039 | 0.0017 | 0.0254 | 0.1383 | 0.0521 | Objective socioeconomic/environmental |
| Alzheimer's disease | Father not alive | 296617 | 0.0085 | 0.0016 | 5.90e-8 | 9.64e-7 | 2.30e-7 | Objective socioeconomic/environmental |
| Alzheimer's disease | Mother not alive | 299550 | 0.0164 | 0.0015 | 1.64e-26 | 8.04e-25 | 1.48e-25 | Objective socioeconomic/environmental |
| Alzheimer's disease | Non-accidental death in close genetic family | 95591 | 0.0013 | 0.0032 | 0.6820 | 0.8318 | 0.7938 | Objective life events |
| Alzheimer's disease | Death of a close relative | 304634 | -0.0011 | 0.0018 | 0.5450 | 0.8081 | 0.6867 | Objective life events |
| Alzheimer's disease | Death of spouse or partner | 304634 | 0.0016 | 0.0018 | 0.3606 | 0.8081 | 0.5016 | Objective life events |
| Alzheimer's disease | Serious illness/injury/assault of close relative | 304634 | -0.0012 | 0.0018 | 0.5139 | 0.8081 | 0.6570 | Objective life events |
| Alzheimer's disease | Infrequent friend/family visits | 302589 | -0.0015 | 0.0018 | 0.3848 | 0.8081 | 0.5270 | Objective socioeconomic/environmental |
| Alzheimer's disease | Loneliness/isolation | 300002 | 0.0013 | 0.0018 | 0.4926 | 0.8081 | 0.6334 | Perception/subjectivity |
| Alzheimer's disease | Social isolation behaviour | 304634 | -0.0019 | 0.0018 | 0.2977 | 0.7676 | 0.4304 | Behavioural/lifestyle |
| ADHD | Smoking | 303330 | 0.0569 | 0.0018 | 1.00e-220 | 4.91e-219 | 1.47e-218 | Behavioural/lifestyle |
| ADHD | Frequent alcohol intake | 304278 | -0.0306 | 0.0018 | 2.17e-66 | 1.18e-65 | 3.99e-65 | Behavioural/lifestyle |
| ADHD | Low moderate physical activity | 290819 | 0.0194 | 0.0018 | 8.80e-26 | 3.08e-25 | 7.77e-25 | Behavioural/lifestyle |
| ADHD | Low vigorous physical activity | 290787 | 0.0149 | 0.0018 | 5.77e-16 | 1.49e-15 | 3.53e-15 | Behavioural/lifestyle |
| ADHD | Low vegetable consumption | 43333 | 0.0183 | 0.0048 | 0.0001 | 0.0002 | 0.0004 | Behavioural/lifestyle |
| ADHD | Low fish consumption | 43333 | 0.0033 | 0.0048 | 0.4907 | 0.5009 | 0.6328 | Behavioural/lifestyle |
| ADHD | Low fruit consumption | 43333 | 0.0219 | 0.0047 | 3.51e-6 | 5.38e-6 | 1.17e-5 | Behavioural/lifestyle |
| ADHD | Low yogurt/ice cream consumption | 43333 | 0.0050 | 0.0048 | 0.2967 | 0.3094 | 0.4304 | Behavioural/lifestyle |
| ADHD | Ever taken cannabis | 83055 | 0.0142 | 0.0033 | 1.55e-5 | 2.16e-5 | 4.77e-5 | Behavioural/lifestyle |
| ADHD | Marital separation or divorce | 304634 | 0.0083 | 0.0018 | 3.94e-6 | 5.85e-6 | 1.31e-5 | Objective life events |
| ADHD | Sexual interference by partner/ex-partner without consent | 82967 | 0.0103 | 0.0035 | 0.0032 | 0.0040 | 0.0076 | Interpersonal adversity/victimisation |
| ADHD | Victim of sexual assault | 82369 | 0.0155 | 0.0034 | 7.16e-6 | 1.03e-5 | 2.30e-5 | Interpersonal adversity/victimisation |
| ADHD | Belittlement by partner/ex-partner | 82956 | 0.0233 | 0.0034 | 8.41e-12 | 1.96e-11 | 4.31e-11 | Perception/subjectivity |
| ADHD | Physical violence by partner/ex-partner | 82976 | 0.0338 | 0.0034 | 1.02e-22 | 3.12e-22 | 8.32e-22 | Interpersonal adversity/victimisation |
| ADHD | Victim of physically violent crime | 83062 | 0.0065 | 0.0034 | 0.0568 | 0.0618 | 0.1079 | Interpersonal adversity/victimisation |
| ADHD | Felt hated by family member as a child | 83007 | 0.0335 | 0.0034 | 2.75e-23 | 8.97e-23 | 2.33e-22 | Perception/subjectivity |
| ADHD | Physically abused by family as a child | 83033 | 0.0478 | 0.0034 | 2.36e-44 | 1.05e-43 | 3.59e-43 | Interpersonal adversity/victimisation |
| ADHD | Sexually molested as a child | 82479 | 0.0222 | 0.0035 | 1.85e-10 | 3.95e-10 | 8.79e-10 | Interpersonal adversity/victimisation |
| ADHD | Low family relationship satisfaction | 99904 | 0.0117 | 0.0032 | 0.0002 | 0.0003 | 0.0006 | Perception/subjectivity |
| ADHD | Combat or war-zone exposure | 83097 | 0.0185 | 0.0034 | 5.67e-8 | 9.91e-8 | 2.23e-7 | Objective life events |
| ADHD | Financial difficulties | 304634 | 0.0316 | 0.0018 | 8.38e-71 | 5.87e-70 | 1.85e-69 | Objective socioeconomic/environmental |
| ADHD | Low financial situation satisfaction | 100315 | 0.0394 | 0.0031 | 1.43e-36 | 5.38e-36 | 1.61e-35 | Perception/subjectivity |
| ADHD | Not in paid employment/self-employment | 304634 | 0.0032 | 0.0015 | 0.0330 | 0.0367 | 0.0658 | Objective socioeconomic/environmental |
| ADHD | Unable to work because of sickness/disability | 304634 | 0.0275 | 0.0018 | 2.61e-50 | 1.28e-49 | 4.27e-49 | Objective socioeconomic/environmental |
| ADHD | Unemployed | 304634 | 0.0106 | 0.0018 | 6.51e-9 | 1.20e-8 | 2.73e-8 | Objective socioeconomic/environmental |
| ADHD | Unable to pay rent/mortgage as an adult | 81997 | 0.0189 | 0.0035 | 6.71e-8 | 1.13e-7 | 2.59e-7 | Objective socioeconomic/environmental |
| ADHD | Non-owner accommodation / renting | 300624 | 0.0298 | 0.0017 | 1.11e-69 | 6.80e-69 | 2.33e-68 | Objective socioeconomic/environmental |
| ADHD | Low average household income | 261733 | 0.0428 | 0.0018 | 1.45e-130 | 2.37e-129 | 6.41e-129 | Objective socioeconomic/environmental |
| ADHD | Higher PM10 air pollution | 277926 | 0.0114 | 0.0018 | 5.69e-10 | 1.12e-9 | 2.56e-9 | Objective socioeconomic/environmental |
| ADHD | Higher PM2.5 air pollution | 277926 | 0.0126 | 0.0017 | 5.47e-13 | 1.34e-12 | 2.98e-12 | Objective socioeconomic/environmental |
| ADHD | Maternal smoking around birth | 261771 | 0.0423 | 0.0019 | 5.07e-105 | 6.21e-104 | 1.72e-103 | Behavioural/lifestyle |
| ADHD | Higher nitrogen dioxide air pollution | 300348 | 0.0091 | 0.0016 | 6.63e-9 | 1.20e-8 | 2.76e-8 | Objective socioeconomic/environmental |
| ADHD | Higher Townsend deprivation | 304310 | 0.0346 | 0.0017 | 6.83e-95 | 6.70e-94 | 2.15e-93 | Objective socioeconomic/environmental |
| ADHD | Urban/rural population density | 301809 | -0.0059 | 0.0012 | 6.41e-7 | 1.01e-6 | 2.30e-6 | Objective socioeconomic/environmental |
| ADHD | Low happiness with own health | 83016 | 0.0217 | 0.0035 | 5.29e-10 | 1.08e-9 | 2.43e-9 | Perception/subjectivity |
| ADHD | Poor overall health rating | 303427 | 0.0480 | 0.0018 | 1.49e-156 | 3.66e-155 | 9.41e-155 | Perception/subjectivity |
| ADHD | Serious life-threatening accident | 83098 | 0.0183 | 0.0034 | 7.99e-8 | 1.31e-7 | 3.04e-7 | Objective life events |
| ADHD | Diagnosed with life-threatening illness | 82917 | 0.0138 | 0.0035 | 6.60e-5 | 8.99e-5 | 0.0002 | Objective life events |
| ADHD | Serious illness/injury/assault to self | 304634 | 0.0161 | 0.0018 | 5.27e-19 | 1.52e-18 | 3.63e-18 | Objective life events |
| ADHD | Poor health sum score | 304634 | 0.0325 | 0.0017 | 2.98e-77 | 2.43e-76 | 8.22e-76 | Objective socioeconomic/environmental |
| ADHD | Father not alive | 296617 | 0.0026 | 0.0016 | 0.0990 | 0.1055 | 0.1790 | Objective socioeconomic/environmental |
| ADHD | Mother not alive | 299550 | 0.0035 | 0.0016 | 0.0261 | 0.0298 | 0.0533 | Objective socioeconomic/environmental |
| ADHD | Non-accidental death in close genetic family | 95591 | 0.0079 | 0.0032 | 0.0145 | 0.0169 | 0.0307 | Objective life events |
| ADHD | Death of a close relative | 304634 | 0.0059 | 0.0018 | 0.0012 | 0.0015 | 0.0030 | Objective life events |
| ADHD | Death of spouse or partner | 304634 | 0.0006 | 0.0018 | 0.7304 | 0.7304 | 0.8302 | Objective life events |
| ADHD | Serious illness/injury/assault of close relative | 304634 | -0.0049 | 0.0018 | 0.0073 | 0.0088 | 0.0160 | Objective life events |
| ADHD | Infrequent friend/family visits | 302589 | -0.0118 | 0.0018 | 3.78e-11 | 8.42e-11 | 1.85e-10 | Objective socioeconomic/environmental |
| ADHD | Loneliness/isolation | 300002 | 0.0243 | 0.0018 | 1.01e-40 | 4.14e-40 | 1.31e-39 | Perception/subjectivity |
| ADHD | Social isolation behaviour | 304634 | 0.0150 | 0.0018 | 7.81e-17 | 2.13e-16 | 4.99e-16 | Behavioural/lifestyle |
| Bipolar disorder | Smoking | 303330 | 0.0155 | 0.0018 | 1.06e-17 | 5.19e-16 | 7.07e-17 | Behavioural/lifestyle |
| Bipolar disorder | Frequent alcohol intake | 304278 | 0.0056 | 0.0018 | 0.0018 | 0.0055 | 0.0043 | Behavioural/lifestyle |
| Bipolar disorder | Low moderate physical activity | 290819 | 0.0152 | 0.0019 | 4.01e-16 | 6.55e-15 | 2.49e-15 | Behavioural/lifestyle |
| Bipolar disorder | Low vigorous physical activity | 290787 | 0.0124 | 0.0019 | 2.04e-11 | 2.00e-10 | 1.01e-10 | Behavioural/lifestyle |
| Bipolar disorder | Low vegetable consumption | 43333 | -0.0061 | 0.0048 | 0.2072 | 0.3627 | 0.3293 | Behavioural/lifestyle |
| Bipolar disorder | Low fish consumption | 43333 | -0.0020 | 0.0048 | 0.6793 | 0.7896 | 0.7938 | Behavioural/lifestyle |
| Bipolar disorder | Low fruit consumption | 43333 | -0.0053 | 0.0048 | 0.2661 | 0.4205 | 0.3964 | Behavioural/lifestyle |
| Bipolar disorder | Low yogurt/ice cream consumption | 43333 | -0.0018 | 0.0048 | 0.7090 | 0.7896 | 0.8122 | Behavioural/lifestyle |
| Bipolar disorder | Ever taken cannabis | 83055 | 0.0256 | 0.0033 | 6.14e-15 | 7.52e-14 | 3.52e-14 | Behavioural/lifestyle |
| Bipolar disorder | Marital separation or divorce | 304634 | 0.0071 | 0.0018 | 8.30e-5 | 0.0004 | 0.0002 | Objective life events |
| Bipolar disorder | Sexual interference by partner/ex-partner without consent | 82967 | 0.0066 | 0.0033 | 0.0460 | 0.1026 | 0.0887 | Interpersonal adversity/victimisation |
| Bipolar disorder | Victim of sexual assault | 82369 | 0.0221 | 0.0034 | 8.12e-11 | 6.63e-10 | 3.94e-10 | Interpersonal adversity/victimisation |
| Bipolar disorder | Belittlement by partner/ex-partner | 82956 | 0.0094 | 0.0034 | 0.0061 | 0.0177 | 0.0138 | Perception/subjectivity |
| Bipolar disorder | Physical violence by partner/ex-partner | 82976 | 0.0071 | 0.0035 | 0.0391 | 0.0912 | 0.0762 | Interpersonal adversity/victimisation |
| Bipolar disorder | Victim of physically violent crime | 83062 | 0.0138 | 0.0034 | 6.28e-5 | 0.0003 | 0.0002 | Interpersonal adversity/victimisation |
| Bipolar disorder | Felt hated by family member as a child | 83007 | 0.0113 | 0.0034 | 0.0010 | 0.0038 | 0.0026 | Perception/subjectivity |
| Bipolar disorder | Physically abused by family as a child | 83033 | 0.0001 | 0.0035 | 0.9733 | 0.9936 | 0.9913 | Interpersonal adversity/victimisation |
| Bipolar disorder | Sexually molested as a child | 82479 | 0.0159 | 0.0034 | 3.42e-6 | 2.09e-5 | 1.15e-5 | Interpersonal adversity/victimisation |
| Bipolar disorder | Low family relationship satisfaction | 99904 | 0.0269 | 0.0032 | 2.94e-17 | 7.21e-16 | 1.94e-16 | Perception/subjectivity |
| Bipolar disorder | Combat or war-zone exposure | 83097 | 0.0019 | 0.0034 | 0.5743 | 0.7405 | 0.7154 | Objective life events |
| Bipolar disorder | Financial difficulties | 304634 | 0.0071 | 0.0018 | 6.60e-5 | 0.0003 | 0.0002 | Objective socioeconomic/environmental |
| Bipolar disorder | Low financial situation satisfaction | 100315 | 0.0037 | 0.0032 | 0.2496 | 0.4077 | 0.3757 | Perception/subjectivity |
| Bipolar disorder | Not in paid employment/self-employment | 304634 | -0.0007 | 0.0015 | 0.6283 | 0.7531 | 0.7530 | Objective socioeconomic/environmental |
| Bipolar disorder | Unable to work because of sickness/disability | 304634 | 0.0029 | 0.0018 | 0.1201 | 0.2180 | 0.2119 | Objective socioeconomic/environmental |
| Bipolar disorder | Unemployed | 304634 | 0.0009 | 0.0018 | 0.6036 | 0.7531 | 0.7415 | Objective socioeconomic/environmental |
| Bipolar disorder | Unable to pay rent/mortgage as an adult | 81997 | 0.0013 | 0.0035 | 0.7067 | 0.7896 | 0.8122 | Objective socioeconomic/environmental |
| Bipolar disorder | Non-owner accommodation / renting | 300624 | 0.0057 | 0.0017 | 0.0008 | 0.0033 | 0.0021 | Objective socioeconomic/environmental |
| Bipolar disorder | Low average household income | 261733 | -0.0022 | 0.0018 | 0.2279 | 0.3850 | 0.3525 | Objective socioeconomic/environmental |
| Bipolar disorder | Higher PM10 air pollution | 277926 | -0.0033 | 0.0019 | 0.0714 | 0.1522 | 0.1335 | Objective socioeconomic/environmental |
| Bipolar disorder | Higher PM2.5 air pollution | 277926 | 0.0008 | 0.0018 | 0.6301 | 0.7531 | 0.7531 | Objective socioeconomic/environmental |
| Bipolar disorder | Maternal smoking around birth | 261771 | -0.0006 | 0.0020 | 0.7563 | 0.8236 | 0.8466 | Behavioural/lifestyle |
| Bipolar disorder | Higher nitrogen dioxide air pollution | 300348 | 0.0027 | 0.0016 | 0.0883 | 0.1730 | 0.1622 | Objective socioeconomic/environmental |
| Bipolar disorder | Higher Townsend deprivation | 304310 | 0.0054 | 0.0017 | 0.0014 | 0.0049 | 0.0035 | Objective socioeconomic/environmental |
| Bipolar disorder | Urban/rural population density | 301809 | -5.68e-6 | 0.0012 | 0.9962 | 0.9962 | 0.9995 | Objective socioeconomic/environmental |
| Bipolar disorder | Low happiness with own health | 83016 | 0.0003 | 0.0035 | 0.9221 | 0.9613 | 0.9568 | Perception/subjectivity |
| Bipolar disorder | Poor overall health rating | 303427 | 0.0030 | 0.0018 | 0.0948 | 0.1786 | 0.1727 | Perception/subjectivity |
| Bipolar disorder | Serious life-threatening accident | 83098 | 0.0062 | 0.0035 | 0.0770 | 0.1573 | 0.1433 | Objective life events |
| Bipolar disorder | Diagnosed with life-threatening illness | 82917 | 0.0079 | 0.0034 | 0.0214 | 0.0552 | 0.0447 | Objective life events |
| Bipolar disorder | Serious illness/injury/assault to self | 304634 | 0.0049 | 0.0018 | 0.0075 | 0.0205 | 0.0164 | Objective life events |
| Bipolar disorder | Poor health sum score | 304634 | 0.0004 | 0.0018 | 0.8422 | 0.8971 | 0.9125 | Objective socioeconomic/environmental |
| Bipolar disorder | Father not alive | 296617 | -0.0015 | 0.0016 | 0.3434 | 0.5099 | 0.4885 | Objective socioeconomic/environmental |
| Bipolar disorder | Mother not alive | 299550 | -0.0014 | 0.0016 | 0.3861 | 0.5256 | 0.5272 | Objective socioeconomic/environmental |
| Bipolar disorder | Non-accidental death in close genetic family | 95591 | 0.0073 | 0.0032 | 0.0250 | 0.0612 | 0.0515 | Objective life events |
| Bipolar disorder | Death of a close relative | 304634 | 0.0014 | 0.0018 | 0.4419 | 0.5852 | 0.5852 | Objective life events |
| Bipolar disorder | Death of spouse or partner | 304634 | 0.0016 | 0.0018 | 0.3797 | 0.5256 | 0.5217 | Objective life events |
| Bipolar disorder | Serious illness/injury/assault of close relative | 304634 | 0.0020 | 0.0018 | 0.2800 | 0.4287 | 0.4116 | Objective life events |
| Bipolar disorder | Infrequent friend/family visits | 302589 | -0.0016 | 0.0018 | 0.3598 | 0.5185 | 0.5016 | Objective socioeconomic/environmental |
| Bipolar disorder | Loneliness/isolation | 300002 | 0.0091 | 0.0018 | 7.07e-7 | 4.95e-6 | 2.51e-6 | Perception/subjectivity |
| Bipolar disorder | Social isolation behaviour | 304634 | -0.0057 | 0.0018 | 0.0018 | 0.0055 | 0.0043 | Behavioural/lifestyle |
| Educational attainment | Smoking | 303330 | -0.0512 | 0.0018 | 1.87e-174 | 2.29e-173 | 1.38e-172 | Behavioural/lifestyle |
| Educational attainment | Frequent alcohol intake | 304278 | 0.0549 | 0.0018 | 2.76e-206 | 4.51e-205 | 3.04e-204 | Behavioural/lifestyle |
| Educational attainment | Low moderate physical activity | 290819 | -0.0240 | 0.0019 | 7.07e-38 | 2.67e-37 | 8.66e-37 | Behavioural/lifestyle |
| Educational attainment | Low vigorous physical activity | 290787 | -0.0108 | 0.0019 | 6.33e-9 | 1.11e-8 | 2.68e-8 | Behavioural/lifestyle |
| Educational attainment | Low vegetable consumption | 43333 | -0.0340 | 0.0049 | 3.93e-12 | 8.03e-12 | 2.07e-11 | Behavioural/lifestyle |
| Educational attainment | Low fish consumption | 43333 | -0.0159 | 0.0049 | 0.0011 | 0.0013 | 0.0026 | Behavioural/lifestyle |
| Educational attainment | Low fruit consumption | 43333 | -0.0415 | 0.0048 | 5.45e-18 | 1.21e-17 | 3.70e-17 | Behavioural/lifestyle |
| Educational attainment | Low yogurt/ice cream consumption | 43333 | -0.0075 | 0.0048 | 0.1218 | 0.1326 | 0.2123 | Behavioural/lifestyle |
| Educational attainment | Ever taken cannabis | 83055 | 0.0406 | 0.0033 | 2.06e-34 | 6.30e-34 | 2.21e-33 | Behavioural/lifestyle |
| Educational attainment | Marital separation or divorce | 304634 | -0.0094 | 0.0018 | 2.37e-7 | 3.75e-7 | 8.71e-7 | Objective life events |
| Educational attainment | Sexual interference by partner/ex-partner without consent | 82967 | -0.0019 | 0.0034 | 0.5778 | 0.5898 | 0.7164 | Interpersonal adversity/victimisation |
| Educational attainment | Victim of sexual assault | 82369 | 0.0131 | 0.0035 | 0.0001 | 0.0002 | 0.0004 | Interpersonal adversity/victimisation |
| Educational attainment | Belittlement by partner/ex-partner | 82956 | 0.0051 | 0.0035 | 0.1407 | 0.1498 | 0.2386 | Perception/subjectivity |
| Educational attainment | Physical violence by partner/ex-partner | 82976 | -0.0155 | 0.0035 | 8.17e-6 | 1.21e-5 | 2.61e-5 | Interpersonal adversity/victimisation |
| Educational attainment | Victim of physically violent crime | 83062 | 0.0161 | 0.0035 | 3.26e-6 | 4.99e-6 | 1.10e-5 | Interpersonal adversity/victimisation |
| Educational attainment | Felt hated by family member as a child | 83007 | -0.0038 | 0.0035 | 0.2739 | 0.2855 | 0.4067 | Perception/subjectivity |
| Educational attainment | Physically abused by family as a child | 83033 | -0.0183 | 0.0034 | 9.61e-8 | 1.57e-7 | 3.59e-7 | Interpersonal adversity/victimisation |
| Educational attainment | Sexually molested as a child | 82479 | -0.0083 | 0.0034 | 0.0156 | 0.0182 | 0.0330 | Interpersonal adversity/victimisation |
| Educational attainment | Low family relationship satisfaction | 99904 | 0.0186 | 0.0032 | 3.93e-9 | 7.40e-9 | 1.71e-8 | Perception/subjectivity |
| Educational attainment | Combat or war-zone exposure | 83097 | -0.0094 | 0.0035 | 0.0070 | 0.0084 | 0.0155 | Objective life events |
| Educational attainment | Financial difficulties | 304634 | -0.0244 | 0.0018 | 1.65e-41 | 7.35e-41 | 2.28e-40 | Objective socioeconomic/environmental |
| Educational attainment | Low financial situation satisfaction | 100315 | -0.0401 | 0.0031 | 2.49e-37 | 8.70e-37 | 2.96e-36 | Perception/subjectivity |
| Educational attainment | Not in paid employment/self-employment | 304634 | -0.0125 | 0.0015 | 7.55e-17 | 1.61e-16 | 4.90e-16 | Objective socioeconomic/environmental |
| Educational attainment | Unable to work because of sickness/disability | 304634 | -0.0339 | 0.0019 | 2.19e-73 | 1.34e-72 | 5.38e-72 | Objective socioeconomic/environmental |
| Educational attainment | Unemployed | 304634 | -0.0102 | 0.0018 | 2.71e-8 | 4.57e-8 | 1.10e-7 | Objective socioeconomic/environmental |
| Educational attainment | Unable to pay rent/mortgage as an adult | 81997 | -0.0417 | 0.0035 | 7.80e-32 | 2.25e-31 | 8.00e-31 | Objective socioeconomic/environmental |
| Educational attainment | Non-owner accommodation / renting | 300624 | -0.0352 | 0.0017 | 1.43e-92 | 1.00e-91 | 4.21e-91 | Objective socioeconomic/environmental |
| Educational attainment | Low average household income | 261733 | -0.0942 | 0.0018 | <1e-300 | <1e-300 | <1e-300 | Objective socioeconomic/environmental |
| Educational attainment | Higher PM10 air pollution | 277926 | -0.0241 | 0.0018 | 6.26e-39 | 2.56e-38 | 7.89e-38 | Objective socioeconomic/environmental |
| Educational attainment | Higher PM2.5 air pollution | 277926 | -0.0182 | 0.0018 | 5.23e-25 | 1.35e-24 | 4.52e-24 | Objective socioeconomic/environmental |
| Educational attainment | Maternal smoking around birth | 261771 | -0.0449 | 0.0020 | 9.57e-117 | 7.81e-116 | 3.52e-115 | Behavioural/lifestyle |
| Educational attainment | Higher nitrogen dioxide air pollution | 300348 | -0.0093 | 0.0016 | 5.82e-9 | 1.06e-8 | 2.52e-8 | Objective socioeconomic/environmental |
| Educational attainment | Higher Townsend deprivation | 304310 | -0.0422 | 0.0017 | 5.85e-137 | 5.73e-136 | 2.87e-135 | Objective socioeconomic/environmental |
| Educational attainment | Urban/rural population density | 301809 | 0.0106 | 0.0012 | 3.40e-19 | 7.94e-19 | 2.38e-18 | Objective socioeconomic/environmental |
| Educational attainment | Low happiness with own health | 83016 | -0.0142 | 0.0035 | 5.74e-5 | 7.81e-5 | 0.0002 | Perception/subjectivity |
| Educational attainment | Poor overall health rating | 303427 | -0.0676 | 0.0018 | 1.27e-300 | 3.11e-299 | 2.80e-298 | Perception/subjectivity |
| Educational attainment | Serious life-threatening accident | 83098 | 0.0100 | 0.0035 | 0.0045 | 0.0055 | 0.0102 | Objective life events |
| Educational attainment | Diagnosed with life-threatening illness | 82917 | -0.0082 | 0.0035 | 0.0188 | 0.0214 | 0.0394 | Objective life events |
| Educational attainment | Serious illness/injury/assault to self | 304634 | -0.0068 | 0.0018 | 0.0002 | 0.0003 | 0.0006 | Objective life events |
| Educational attainment | Poor health sum score | 304634 | -0.0310 | 0.0018 | 1.87e-68 | 1.02e-67 | 3.75e-67 | Objective socioeconomic/environmental |
| Educational attainment | Father not alive | 296617 | -0.0070 | 0.0016 | 1.12e-5 | 1.61e-5 | 3.54e-5 | Objective socioeconomic/environmental |
| Educational attainment | Mother not alive | 299550 | -0.0067 | 0.0016 | 1.89e-5 | 2.64e-5 | 5.70e-5 | Objective socioeconomic/environmental |
| Educational attainment | Non-accidental death in close genetic family | 95591 | -0.0015 | 0.0033 | 0.6415 | 0.6415 | 0.7625 | Objective life events |
| Educational attainment | Death of a close relative | 304634 | -0.0114 | 0.0018 | 5.69e-10 | 1.11e-9 | 2.56e-9 | Objective life events |
| Educational attainment | Death of spouse or partner | 304634 | -0.0030 | 0.0018 | 0.0988 | 0.1100 | 0.1790 | Objective life events |
| Educational attainment | Serious illness/injury/assault of close relative | 304634 | 0.0167 | 0.0018 | 4.88e-20 | 1.20e-19 | 3.59e-19 | Objective life events |
| Educational attainment | Infrequent friend/family visits | 302589 | 0.0249 | 0.0018 | 5.16e-44 | 2.53e-43 | 7.58e-43 | Objective socioeconomic/environmental |
| Educational attainment | Loneliness/isolation | 300002 | -0.0230 | 0.0018 | 7.08e-36 | 2.31e-35 | 7.81e-35 | Perception/subjectivity |
| Educational attainment | Social isolation behaviour | 304634 | -0.0195 | 0.0018 | 2.90e-27 | 7.89e-27 | 2.72e-26 | Behavioural/lifestyle |
| Extraversion | Smoking | 303330 | 0.0079 | 0.0018 | 1.27e-5 | 0.0002 | 3.99e-5 | Behavioural/lifestyle |
| Extraversion | Frequent alcohol intake | 304278 | 0.0016 | 0.0018 | 0.3636 | 0.6132 | 0.5042 | Behavioural/lifestyle |
| Extraversion | Low moderate physical activity | 290819 | 0.0028 | 0.0019 | 0.1259 | 0.3987 | 0.2185 | Behavioural/lifestyle |
| Extraversion | Low vigorous physical activity | 290787 | 0.0050 | 0.0019 | 0.0070 | 0.0341 | 0.0155 | Behavioural/lifestyle |
| Extraversion | Low vegetable consumption | 43333 | 0.0064 | 0.0049 | 0.1871 | 0.4223 | 0.3025 | Behavioural/lifestyle |
| Extraversion | Low fish consumption | 43333 | -0.0058 | 0.0048 | 0.2280 | 0.4468 | 0.3525 | Behavioural/lifestyle |
| Extraversion | Low fruit consumption | 43333 | 0.0076 | 0.0048 | 0.1088 | 0.3987 | 0.1951 | Behavioural/lifestyle |
| Extraversion | Low yogurt/ice cream consumption | 43333 | -0.0014 | 0.0048 | 0.7679 | 0.9374 | 0.8513 | Behavioural/lifestyle |
| Extraversion | Ever taken cannabis | 83055 | -0.0046 | 0.0033 | 0.1633 | 0.4223 | 0.2708 | Behavioural/lifestyle |
| Extraversion | Marital separation or divorce | 304634 | 0.0027 | 0.0018 | 0.1302 | 0.3987 | 0.2231 | Objective life events |
| Extraversion | Sexual interference by partner/ex-partner without consent | 82967 | -0.0026 | 0.0034 | 0.4469 | 0.6636 | 0.5901 | Interpersonal adversity/victimisation |
| Extraversion | Victim of sexual assault | 82369 | -0.0005 | 0.0035 | 0.8879 | 0.9834 | 0.9435 | Interpersonal adversity/victimisation |
| Extraversion | Belittlement by partner/ex-partner | 82956 | -0.0046 | 0.0034 | 0.1817 | 0.4223 | 0.2989 | Perception/subjectivity |
| Extraversion | Physical violence by partner/ex-partner | 82976 | 0.0010 | 0.0035 | 0.7844 | 0.9374 | 0.8614 | Interpersonal adversity/victimisation |
| Extraversion | Victim of physically violent crime | 83062 | 0.0011 | 0.0034 | 0.7458 | 0.9374 | 0.8412 | Interpersonal adversity/victimisation |
| Extraversion | Felt hated by family member as a child | 83007 | -0.0043 | 0.0034 | 0.2076 | 0.4360 | 0.3293 | Perception/subjectivity |
| Extraversion | Physically abused by family as a child | 83033 | -0.0003 | 0.0034 | 0.9367 | 0.9834 | 0.9689 | Interpersonal adversity/victimisation |
| Extraversion | Sexually molested as a child | 82479 | -0.0027 | 0.0035 | 0.4342 | 0.6636 | 0.5776 | Interpersonal adversity/victimisation |
| Extraversion | Low family relationship satisfaction | 99904 | -0.0121 | 0.0032 | 0.0001 | 0.0009 | 0.0004 | Perception/subjectivity |
| Extraversion | Combat or war-zone exposure | 83097 | 0.0043 | 0.0034 | 0.2135 | 0.4360 | 0.3363 | Objective life events |
| Extraversion | Financial difficulties | 304634 | 0.0070 | 0.0018 | 0.0001 | 0.0008 | 0.0003 | Objective socioeconomic/environmental |
| Extraversion | Low financial situation satisfaction | 100315 | -0.0016 | 0.0031 | 0.6154 | 0.8869 | 0.7491 | Perception/subjectivity |
| Extraversion | Not in paid employment/self-employment | 304634 | -0.0005 | 0.0015 | 0.7114 | 0.9374 | 0.8124 | Objective socioeconomic/environmental |
| Extraversion | Unable to work because of sickness/disability | 304634 | 0.0076 | 0.0018 | 2.48e-5 | 0.0002 | 7.44e-5 | Objective socioeconomic/environmental |
| Extraversion | Unemployed | 304634 | 0.0005 | 0.0018 | 0.7790 | 0.9374 | 0.8588 | Objective socioeconomic/environmental |
| Extraversion | Unable to pay rent/mortgage as an adult | 81997 | 0.0046 | 0.0035 | 0.1873 | 0.4223 | 0.3025 | Objective socioeconomic/environmental |
| Extraversion | Non-owner accommodation / renting | 300624 | 0.0056 | 0.0017 | 0.0010 | 0.0056 | 0.0026 | Objective socioeconomic/environmental |
| Extraversion | Low average household income | 261733 | -0.0023 | 0.0018 | 0.1896 | 0.4223 | 0.3052 | Objective socioeconomic/environmental |
| Extraversion | Higher PM10 air pollution | 277926 | 0.0004 | 0.0018 | 0.8408 | 0.9809 | 0.9125 | Objective socioeconomic/environmental |
| Extraversion | Higher PM2.5 air pollution | 277926 | -0.0015 | 0.0017 | 0.3754 | 0.6132 | 0.5174 | Objective socioeconomic/environmental |
| Extraversion | Maternal smoking around birth | 261771 | 0.0036 | 0.0019 | 0.0676 | 0.2760 | 0.1269 | Behavioural/lifestyle |
| Extraversion | Higher nitrogen dioxide air pollution | 300348 | -0.0024 | 0.0016 | 0.1274 | 0.3987 | 0.2203 | Objective socioeconomic/environmental |
| Extraversion | Higher Townsend deprivation | 304310 | 6.36e-5 | 0.0017 | 0.9698 | 0.9834 | 0.9906 | Objective socioeconomic/environmental |
| Extraversion | Urban/rural population density | 301809 | 0.0010 | 0.0012 | 0.4142 | 0.6548 | 0.5536 | Objective socioeconomic/environmental |
| Extraversion | Low happiness with own health | 83016 | -0.0014 | 0.0035 | 0.6822 | 0.9286 | 0.7938 | Perception/subjectivity |
| Extraversion | Poor overall health rating | 303427 | -3.75e-5 | 0.0018 | 0.9834 | 0.9834 | 0.9939 | Perception/subjectivity |
| Extraversion | Serious life-threatening accident | 83098 | 0.0040 | 0.0035 | 0.2480 | 0.4673 | 0.3745 | Objective life events |
| Extraversion | Diagnosed with life-threatening illness | 82917 | 0.0004 | 0.0035 | 0.9146 | 0.9834 | 0.9530 | Objective life events |
| Extraversion | Serious illness/injury/assault to self | 304634 | 0.0039 | 0.0018 | 0.0338 | 0.1505 | 0.0671 | Objective life events |
| Extraversion | Poor health sum score | 304634 | 0.0089 | 0.0017 | 3.92e-7 | 6.41e-6 | 1.43e-6 | Objective socioeconomic/environmental |
| Extraversion | Father not alive | 296617 | 6.24e-5 | 0.0016 | 0.9684 | 0.9834 | 0.9906 | Objective socioeconomic/environmental |
| Extraversion | Mother not alive | 299550 | -0.0014 | 0.0016 | 0.3745 | 0.6132 | 0.5174 | Objective socioeconomic/environmental |
| Extraversion | Non-accidental death in close genetic family | 95591 | -0.0014 | 0.0032 | 0.6559 | 0.9183 | 0.7755 | Objective life events |
| Extraversion | Death of a close relative | 304634 | 0.0019 | 0.0018 | 0.2945 | 0.5344 | 0.4286 | Objective life events |
| Extraversion | Death of spouse or partner | 304634 | 0.0002 | 0.0018 | 0.9016 | 0.9834 | 0.9489 | Objective life events |
| Extraversion | Serious illness/injury/assault of close relative | 304634 | 0.0024 | 0.0018 | 0.1847 | 0.4223 | 0.3021 | Objective life events |
| Extraversion | Infrequent friend/family visits | 302589 | -0.0142 | 0.0018 | 1.74e-15 | 4.26e-14 | 1.04e-14 | Objective socioeconomic/environmental |
| Extraversion | Loneliness/isolation | 300002 | -0.0070 | 0.0018 | 0.0001 | 0.0009 | 0.0004 | Perception/subjectivity |
| Extraversion | Social isolation behaviour | 304634 | -0.0174 | 0.0018 | 5.43e-22 | 2.66e-20 | 4.27e-21 | Behavioural/lifestyle |
| LDL cholesterol | Smoking | 303330 | -0.0039 | 0.0018 | 0.0284 | 0.1580 | 0.0574 | Behavioural/lifestyle |
| LDL cholesterol | Frequent alcohol intake | 304278 | -0.0009 | 0.0018 | 0.6166 | 0.8310 | 0.7491 | Behavioural/lifestyle |
| LDL cholesterol | Low moderate physical activity | 290819 | 0.0046 | 0.0018 | 0.0128 | 0.0894 | 0.0272 | Behavioural/lifestyle |
| LDL cholesterol | Low vigorous physical activity | 290787 | 0.0027 | 0.0018 | 0.1406 | 0.4592 | 0.2386 | Behavioural/lifestyle |
| LDL cholesterol | Low vegetable consumption | 43333 | 0.0005 | 0.0048 | 0.9163 | 0.9552 | 0.9530 | Behavioural/lifestyle |
| LDL cholesterol | Low fish consumption | 43333 | -0.0013 | 0.0048 | 0.7853 | 0.8745 | 0.8614 | Behavioural/lifestyle |
| LDL cholesterol | Low fruit consumption | 43333 | -0.0021 | 0.0047 | 0.6642 | 0.8345 | 0.7827 | Behavioural/lifestyle |
| LDL cholesterol | Low yogurt/ice cream consumption | 43333 | -0.0040 | 0.0048 | 0.4000 | 0.6758 | 0.5378 | Behavioural/lifestyle |
| LDL cholesterol | Ever taken cannabis | 83055 | 0.0040 | 0.0033 | 0.2214 | 0.5469 | 0.3462 | Behavioural/lifestyle |
| LDL cholesterol | Marital separation or divorce | 304634 | 0.0008 | 0.0018 | 0.6444 | 0.8310 | 0.7640 | Objective life events |
| LDL cholesterol | Sexual interference by partner/ex-partner without consent | 82967 | 0.0030 | 0.0035 | 0.3894 | 0.6758 | 0.5301 | Interpersonal adversity/victimisation |
| LDL cholesterol | Victim of sexual assault | 82369 | 0.0034 | 0.0034 | 0.3259 | 0.6674 | 0.4666 | Interpersonal adversity/victimisation |
| LDL cholesterol | Belittlement by partner/ex-partner | 82956 | -0.0032 | 0.0034 | 0.3472 | 0.6674 | 0.4908 | Perception/subjectivity |
| LDL cholesterol | Physical violence by partner/ex-partner | 82976 | -0.0004 | 0.0035 | 0.9124 | 0.9552 | 0.9530 | Interpersonal adversity/victimisation |
| LDL cholesterol | Victim of physically violent crime | 83062 | 0.0025 | 0.0034 | 0.4674 | 0.7387 | 0.6116 | Interpersonal adversity/victimisation |
| LDL cholesterol | Felt hated by family member as a child | 83007 | -0.0024 | 0.0035 | 0.4856 | 0.7435 | 0.6280 | Perception/subjectivity |
| LDL cholesterol | Physically abused by family as a child | 83033 | -0.0044 | 0.0035 | 0.2023 | 0.5469 | 0.3244 | Interpersonal adversity/victimisation |
| LDL cholesterol | Sexually molested as a child | 82479 | 0.0032 | 0.0035 | 0.3541 | 0.6674 | 0.4958 | Interpersonal adversity/victimisation |
| LDL cholesterol | Low family relationship satisfaction | 99904 | 0.0032 | 0.0031 | 0.3098 | 0.6674 | 0.4465 | Perception/subjectivity |
| LDL cholesterol | Combat or war-zone exposure | 83097 | -0.0020 | 0.0035 | 0.5586 | 0.8026 | 0.7018 | Objective life events |
| LDL cholesterol | Financial difficulties | 304634 | 0.0001 | 0.0018 | 0.9404 | 0.9600 | 0.9689 | Objective socioeconomic/environmental |
| LDL cholesterol | Low financial situation satisfaction | 100315 | 0.0064 | 0.0031 | 0.0378 | 0.1854 | 0.0742 | Perception/subjectivity |
| LDL cholesterol | Not in paid employment/self-employment | 304634 | 0.0023 | 0.0015 | 0.1187 | 0.4159 | 0.2113 | Objective socioeconomic/environmental |
| LDL cholesterol | Unable to work because of sickness/disability | 304634 | 0.0017 | 0.0018 | 0.3450 | 0.6674 | 0.4893 | Objective socioeconomic/environmental |
| LDL cholesterol | Unemployed | 304634 | -0.0022 | 0.0018 | 0.2131 | 0.5469 | 0.3363 | Objective socioeconomic/environmental |
| LDL cholesterol | Unable to pay rent/mortgage as an adult | 81997 | -0.0010 | 0.0035 | 0.7671 | 0.8742 | 0.8513 | Objective socioeconomic/environmental |
| LDL cholesterol | Non-owner accommodation / renting | 300624 | 0.0034 | 0.0017 | 0.0454 | 0.2023 | 0.0879 | Objective socioeconomic/environmental |
| LDL cholesterol | Low average household income | 261733 | 0.0067 | 0.0018 | 0.0001 | 0.0014 | 0.0004 | Objective socioeconomic/environmental |
| LDL cholesterol | Higher PM10 air pollution | 277926 | -0.0004 | 0.0018 | 0.8233 | 0.8965 | 0.8965 | Objective socioeconomic/environmental |
| LDL cholesterol | Higher PM2.5 air pollution | 277926 | 0.0006 | 0.0017 | 0.7363 | 0.8590 | 0.8348 | Objective socioeconomic/environmental |
| LDL cholesterol | Maternal smoking around birth | 261771 | 0.0008 | 0.0019 | 0.6929 | 0.8458 | 0.8020 | Behavioural/lifestyle |
| LDL cholesterol | Higher nitrogen dioxide air pollution | 300348 | 0.0019 | 0.0016 | 0.2200 | 0.5469 | 0.3452 | Objective socioeconomic/environmental |
| LDL cholesterol | Higher Townsend deprivation | 304310 | 0.0006 | 0.0017 | 0.7077 | 0.8458 | 0.8122 | Objective socioeconomic/environmental |
| LDL cholesterol | Urban/rural population density | 301809 | -0.0010 | 0.0012 | 0.3919 | 0.6758 | 0.5317 | Objective socioeconomic/environmental |
| LDL cholesterol | Low happiness with own health | 83016 | 0.0076 | 0.0035 | 0.0290 | 0.1580 | 0.0584 | Perception/subjectivity |
| LDL cholesterol | Poor overall health rating | 303427 | 0.0143 | 0.0018 | 2.11e-15 | 5.17e-14 | 1.24e-14 | Perception/subjectivity |
| LDL cholesterol | Serious life-threatening accident | 83098 | -0.0019 | 0.0034 | 0.5733 | 0.8026 | 0.7154 | Objective life events |
| LDL cholesterol | Diagnosed with life-threatening illness | 82917 | 0.0060 | 0.0035 | 0.0876 | 0.3576 | 0.1616 | Objective life events |
| LDL cholesterol | Serious illness/injury/assault to self | 304634 | 0.0028 | 0.0018 | 0.1188 | 0.4159 | 0.2113 | Objective life events |
| LDL cholesterol | Poor health sum score | 304634 | 0.0166 | 0.0017 | 2.31e-21 | 1.13e-19 | 1.79e-20 | Objective socioeconomic/environmental |
| LDL cholesterol | Father not alive | 296617 | 0.0077 | 0.0016 | 9.42e-7 | 1.15e-5 | 3.32e-6 | Objective socioeconomic/environmental |
| LDL cholesterol | Mother not alive | 299550 | 0.0045 | 0.0015 | 0.0034 | 0.0278 | 0.0079 | Objective socioeconomic/environmental |
| LDL cholesterol | Non-accidental death in close genetic family | 95591 | 0.0228 | 0.0032 | 1.48e-12 | 2.41e-11 | 7.84e-12 | Objective life events |
| LDL cholesterol | Death of a close relative | 304634 | -0.0022 | 0.0018 | 0.2301 | 0.5469 | 0.3535 | Objective life events |
| LDL cholesterol | Death of spouse or partner | 304634 | -0.0009 | 0.0018 | 0.6364 | 0.8310 | 0.7585 | Objective life events |
| LDL cholesterol | Serious illness/injury/assault of close relative | 304634 | -4.82e-6 | 0.0018 | 0.9979 | 0.9979 | 0.9995 | Objective life events |
| LDL cholesterol | Infrequent friend/family visits | 302589 | -0.0021 | 0.0018 | 0.2344 | 0.5469 | 0.3589 | Objective socioeconomic/environmental |
| LDL cholesterol | Loneliness/isolation | 300002 | -0.0013 | 0.0018 | 0.4580 | 0.7387 | 0.6029 | Perception/subjectivity |
| LDL cholesterol | Social isolation behaviour | 304634 | 0.0011 | 0.0018 | 0.5226 | 0.7759 | 0.6641 | Behavioural/lifestyle |
| Major depression | Smoking | 303330 | 0.0452 | 0.0018 | 2.47e-138 | 6.06e-137 | 1.36e-136 | Behavioural/lifestyle |
| Major depression | Frequent alcohol intake | 304278 | -0.0194 | 0.0018 | 1.42e-27 | 4.96e-27 | 1.39e-26 | Behavioural/lifestyle |
| Major depression | Low moderate physical activity | 290819 | 0.0199 | 0.0019 | 1.01e-26 | 3.11e-26 | 9.32e-26 | Behavioural/lifestyle |
| Major depression | Low vigorous physical activity | 290787 | 0.0114 | 0.0018 | 5.83e-10 | 1.06e-9 | 2.60e-9 | Behavioural/lifestyle |
| Major depression | Low vegetable consumption | 43333 | -0.0007 | 0.0048 | 0.8780 | 0.8963 | 0.9376 | Behavioural/lifestyle |
| Major depression | Low fish consumption | 43333 | 0.0047 | 0.0048 | 0.3305 | 0.3598 | 0.4716 | Behavioural/lifestyle |
| Major depression | Low fruit consumption | 43333 | 0.0208 | 0.0048 | 1.28e-5 | 1.84e-5 | 3.99e-5 | Behavioural/lifestyle |
| Major depression | Low yogurt/ice cream consumption | 43333 | 0.0104 | 0.0048 | 0.0297 | 0.0338 | 0.0595 | Behavioural/lifestyle |
| Major depression | Ever taken cannabis | 83055 | 0.0156 | 0.0033 | 2.44e-6 | 3.90e-6 | 8.46e-6 | Behavioural/lifestyle |
| Major depression | Marital separation or divorce | 304634 | 0.0124 | 0.0018 | 1.08e-11 | 2.21e-11 | 5.47e-11 | Objective life events |
| Major depression | Sexual interference by partner/ex-partner without consent | 82967 | 0.0149 | 0.0034 | 1.57e-5 | 2.19e-5 | 4.80e-5 | Interpersonal adversity/victimisation |
| Major depression | Victim of sexual assault | 82369 | 0.0235 | 0.0034 | 7.49e-12 | 1.59e-11 | 3.88e-11 | Interpersonal adversity/victimisation |
| Major depression | Belittlement by partner/ex-partner | 82956 | 0.0284 | 0.0034 | 1.40e-16 | 3.62e-16 | 8.83e-16 | Perception/subjectivity |
| Major depression | Physical violence by partner/ex-partner | 82976 | 0.0266 | 0.0035 | 2.33e-14 | 5.43e-14 | 1.32e-13 | Interpersonal adversity/victimisation |
| Major depression | Victim of physically violent crime | 83062 | 0.0144 | 0.0034 | 2.76e-5 | 3.75e-5 | 8.21e-5 | Interpersonal adversity/victimisation |
| Major depression | Felt hated by family member as a child | 83007 | 0.0392 | 0.0035 | 1.45e-29 | 5.47e-29 | 1.46e-28 | Perception/subjectivity |
| Major depression | Physically abused by family as a child | 83033 | 0.0280 | 0.0035 | 1.17e-15 | 2.86e-15 | 7.06e-15 | Interpersonal adversity/victimisation |
| Major depression | Sexually molested as a child | 82479 | 0.0231 | 0.0034 | 1.19e-11 | 2.33e-11 | 5.94e-11 | Interpersonal adversity/victimisation |
| Major depression | Low family relationship satisfaction | 99904 | 0.0404 | 0.0032 | 4.73e-37 | 1.93e-36 | 5.49e-36 | Perception/subjectivity |
| Major depression | Combat or war-zone exposure | 83097 | 0.0179 | 0.0034 | 1.65e-7 | 2.78e-7 | 6.10e-7 | Objective life events |
| Major depression | Financial difficulties | 304634 | 0.0333 | 0.0018 | 5.73e-77 | 7.02e-76 | 1.49e-75 | Objective socioeconomic/environmental |
| Major depression | Low financial situation satisfaction | 100315 | 0.0433 | 0.0031 | 4.31e-43 | 2.11e-42 | 6.13e-42 | Perception/subjectivity |
| Major depression | Not in paid employment/self-employment | 304634 | 0.0061 | 0.0015 | 4.21e-5 | 5.43e-5 | 0.0001 | Objective socioeconomic/environmental |
| Major depression | Unable to work because of sickness/disability | 304634 | 0.0249 | 0.0019 | 6.77e-41 | 3.02e-40 | 9.05e-40 | Objective socioeconomic/environmental |
| Major depression | Unemployed | 304634 | 0.0082 | 0.0018 | 6.84e-6 | 1.02e-5 | 2.23e-5 | Objective socioeconomic/environmental |
| Major depression | Unable to pay rent/mortgage as an adult | 81997 | 0.0187 | 0.0035 | 7.00e-8 | 1.22e-7 | 2.68e-7 | Objective socioeconomic/environmental |
| Major depression | Non-owner accommodation / renting | 300624 | 0.0273 | 0.0017 | 2.25e-58 | 1.38e-57 | 3.82e-57 | Objective socioeconomic/environmental |
| Major depression | Low average household income | 261733 | 0.0320 | 0.0018 | 7.59e-73 | 7.44e-72 | 1.76e-71 | Objective socioeconomic/environmental |
| Major depression | Higher PM10 air pollution | 277926 | 0.0086 | 0.0018 | 3.10e-6 | 4.75e-6 | 1.06e-5 | Objective socioeconomic/environmental |
| Major depression | Higher PM2.5 air pollution | 277926 | 0.0125 | 0.0017 | 7.34e-13 | 1.63e-12 | 3.94e-12 | Objective socioeconomic/environmental |
| Major depression | Maternal smoking around birth | 261771 | 0.0282 | 0.0020 | 3.47e-47 | 1.89e-46 | 5.46e-46 | Behavioural/lifestyle |
| Major depression | Higher nitrogen dioxide air pollution | 300348 | 0.0100 | 0.0016 | 2.22e-10 | 4.18e-10 | 1.04e-9 | Objective socioeconomic/environmental |
| Major depression | Higher Townsend deprivation | 304310 | 0.0293 | 0.0017 | 6.93e-68 | 5.66e-67 | 1.33e-66 | Objective socioeconomic/environmental |
| Major depression | Urban/rural population density | 301809 | -0.0049 | 0.0012 | 3.75e-5 | 4.97e-5 | 0.0001 | Objective socioeconomic/environmental |
| Major depression | Low happiness with own health | 83016 | 0.0325 | 0.0035 | 1.59e-20 | 4.33e-20 | 1.21e-19 | Perception/subjectivity |
| Major depression | Poor overall health rating | 303427 | 0.0523 | 0.0018 | 4.65e-184 | 2.28e-182 | 4.10e-182 | Perception/subjectivity |
| Major depression | Serious life-threatening accident | 83098 | 0.0105 | 0.0035 | 0.0025 | 0.0031 | 0.0060 | Objective life events |
| Major depression | Diagnosed with life-threatening illness | 82917 | 0.0163 | 0.0035 | 2.47e-6 | 3.90e-6 | 8.51e-6 | Objective life events |
| Major depression | Serious illness/injury/assault to self | 304634 | 0.0177 | 0.0018 | 2.38e-22 | 6.87e-22 | 1.91e-21 | Objective life events |
| Major depression | Poor health sum score | 304634 | 0.0291 | 0.0018 | 3.80e-62 | 2.66e-61 | 6.70e-61 | Objective socioeconomic/environmental |
| Major depression | Father not alive | 296617 | 0.0003 | 0.0016 | 0.8703 | 0.8963 | 0.9361 | Objective socioeconomic/environmental |
| Major depression | Mother not alive | 299550 | -0.0008 | 0.0015 | 0.6130 | 0.6530 | 0.7488 | Objective socioeconomic/environmental |
| Major depression | Non-accidental death in close genetic family | 95591 | 0.0089 | 0.0032 | 0.0059 | 0.0070 | 0.0133 | Objective life events |
| Major depression | Death of a close relative | 304634 | 0.0060 | 0.0018 | 0.0009 | 0.0012 | 0.0024 | Objective life events |
| Major depression | Death of spouse or partner | 304634 | 0.0049 | 0.0018 | 0.0070 | 0.0082 | 0.0155 | Objective life events |
| Major depression | Serious illness/injury/assault of close relative | 304634 | 0.0030 | 0.0018 | 0.0998 | 0.1112 | 0.1797 | Objective life events |
| Major depression | Infrequent friend/family visits | 302589 | -2.87e-5 | 0.0018 | 0.9871 | 0.9871 | 0.9939 | Objective socioeconomic/environmental |
| Major depression | Loneliness/isolation | 300002 | 0.0440 | 0.0018 | 3.88e-129 | 6.34e-128 | 1.56e-127 | Perception/subjectivity |
| Major depression | Social isolation behaviour | 304634 | 0.0196 | 0.0018 | 2.04e-27 | 6.67e-27 | 1.96e-26 | Behavioural/lifestyle |
| Neuroticism | Smoking | 303330 | 0.0103 | 0.0018 | 1.10e-8 | 1.34e-7 | 4.52e-8 | Behavioural/lifestyle |
| Neuroticism | Frequent alcohol intake | 304278 | -0.0012 | 0.0018 | 0.5095 | 0.7456 | 0.6532 | Behavioural/lifestyle |
| Neuroticism | Low moderate physical activity | 290819 | 0.0102 | 0.0019 | 3.62e-8 | 3.55e-7 | 1.44e-7 | Behavioural/lifestyle |
| Neuroticism | Low vigorous physical activity | 290787 | 0.0047 | 0.0019 | 0.0108 | 0.0377 | 0.0231 | Behavioural/lifestyle |
| Neuroticism | Low vegetable consumption | 43333 | 0.0014 | 0.0048 | 0.7775 | 0.9071 | 0.8588 | Behavioural/lifestyle |
| Neuroticism | Low fish consumption | 43333 | -0.0015 | 0.0048 | 0.7543 | 0.9071 | 0.8465 | Behavioural/lifestyle |
| Neuroticism | Low fruit consumption | 43333 | 0.0074 | 0.0048 | 0.1214 | 0.2703 | 0.2123 | Behavioural/lifestyle |
| Neuroticism | Low yogurt/ice cream consumption | 43333 | -0.0007 | 0.0048 | 0.8821 | 0.9605 | 0.9396 | Behavioural/lifestyle |
| Neuroticism | Ever taken cannabis | 83055 | 0.0018 | 0.0033 | 0.5903 | 0.8265 | 0.7276 | Behavioural/lifestyle |
| Neuroticism | Marital separation or divorce | 304634 | 0.0027 | 0.0018 | 0.1414 | 0.3012 | 0.2389 | Objective life events |
| Neuroticism | Sexual interference by partner/ex-partner without consent | 82967 | -0.0032 | 0.0035 | 0.3501 | 0.5771 | 0.4933 | Interpersonal adversity/victimisation |
| Neuroticism | Victim of sexual assault | 82369 | 0.0049 | 0.0035 | 0.1599 | 0.3265 | 0.2669 | Interpersonal adversity/victimisation |
| Neuroticism | Belittlement by partner/ex-partner | 82956 | 0.0116 | 0.0034 | 0.0007 | 0.0040 | 0.0019 | Perception/subjectivity |
| Neuroticism | Physical violence by partner/ex-partner | 82976 | 0.0070 | 0.0035 | 0.0447 | 0.1216 | 0.0868 | Interpersonal adversity/victimisation |
| Neuroticism | Victim of physically violent crime | 83062 | 2.15e-6 | 0.0034 | 0.9995 | 0.9995 | 0.9995 | Interpersonal adversity/victimisation |
| Neuroticism | Felt hated by family member as a child | 83007 | 0.0119 | 0.0035 | 0.0007 | 0.0040 | 0.0019 | Perception/subjectivity |
| Neuroticism | Physically abused by family as a child | 83033 | -0.0002 | 0.0035 | 0.9496 | 0.9995 | 0.9762 | Interpersonal adversity/victimisation |
| Neuroticism | Sexually molested as a child | 82479 | 0.0077 | 0.0035 | 0.0284 | 0.0869 | 0.0574 | Interpersonal adversity/victimisation |
| Neuroticism | Low family relationship satisfaction | 99904 | 0.0142 | 0.0032 | 6.76e-6 | 4.81e-5 | 2.22e-5 | Perception/subjectivity |
| Neuroticism | Combat or war-zone exposure | 83097 | 0.0010 | 0.0034 | 0.7607 | 0.9071 | 0.8493 | Objective life events |
| Neuroticism | Financial difficulties | 304634 | 0.0005 | 0.0018 | 0.7683 | 0.9071 | 0.8513 | Objective socioeconomic/environmental |
| Neuroticism | Low financial situation satisfaction | 100315 | 0.0098 | 0.0031 | 0.0017 | 0.0075 | 0.0041 | Perception/subjectivity |
| Neuroticism | Not in paid employment/self-employment | 304634 | 0.0018 | 0.0015 | 0.2268 | 0.4117 | 0.3525 | Objective socioeconomic/environmental |
| Neuroticism | Unable to work because of sickness/disability | 304634 | 0.0013 | 0.0018 | 0.4707 | 0.7208 | 0.6123 | Objective socioeconomic/environmental |
| Neuroticism | Unemployed | 304634 | 6.71e-5 | 0.0018 | 0.9704 | 0.9995 | 0.9906 | Objective socioeconomic/environmental |
| Neuroticism | Unable to pay rent/mortgage as an adult | 81997 | -0.0009 | 0.0035 | 0.7964 | 0.9075 | 0.8715 | Objective socioeconomic/environmental |
| Neuroticism | Non-owner accommodation / renting | 300624 | 0.0030 | 0.0017 | 0.0778 | 0.1906 | 0.1442 | Objective socioeconomic/environmental |
| Neuroticism | Low average household income | 261733 | 0.0141 | 0.0018 | 2.20e-15 | 3.59e-14 | 1.28e-14 | Objective socioeconomic/environmental |
| Neuroticism | Higher PM10 air pollution | 277926 | 0.0050 | 0.0018 | 0.0067 | 0.0254 | 0.0151 | Objective socioeconomic/environmental |
| Neuroticism | Higher PM2.5 air pollution | 277926 | 0.0016 | 0.0017 | 0.3533 | 0.5771 | 0.4958 | Objective socioeconomic/environmental |
| Neuroticism | Maternal smoking around birth | 261771 | 0.0022 | 0.0019 | 0.2661 | 0.4656 | 0.3964 | Behavioural/lifestyle |
| Neuroticism | Higher nitrogen dioxide air pollution | 300348 | 0.0021 | 0.0016 | 0.1816 | 0.3499 | 0.2989 | Objective socioeconomic/environmental |
| Neuroticism | Higher Townsend deprivation | 304310 | 0.0047 | 0.0017 | 0.0045 | 0.0183 | 0.0102 | Objective socioeconomic/environmental |
| Neuroticism | Urban/rural population density | 301809 | -0.0027 | 0.0012 | 0.0242 | 0.0791 | 0.0501 | Objective socioeconomic/environmental |
| Neuroticism | Low happiness with own health | 83016 | 0.0114 | 0.0035 | 0.0010 | 0.0051 | 0.0026 | Perception/subjectivity |
| Neuroticism | Poor overall health rating | 303427 | 0.0166 | 0.0018 | 3.62e-20 | 1.77e-18 | 2.70e-19 | Perception/subjectivity |
| Neuroticism | Serious life-threatening accident | 83098 | 0.0005 | 0.0034 | 0.8755 | 0.9605 | 0.9371 | Objective life events |
| Neuroticism | Diagnosed with life-threatening illness | 82917 | 7.62e-5 | 0.0034 | 0.9822 | 0.9995 | 0.9939 | Objective life events |
| Neuroticism | Serious illness/injury/assault to self | 304634 | 0.0008 | 0.0018 | 0.6656 | 0.8814 | 0.7827 | Objective life events |
| Neuroticism | Poor health sum score | 304634 | 0.0034 | 0.0017 | 0.0540 | 0.1393 | 0.1031 | Objective socioeconomic/environmental |
| Neuroticism | Father not alive | 296617 | -0.0006 | 0.0016 | 0.6882 | 0.8874 | 0.7986 | Objective socioeconomic/environmental |
| Neuroticism | Mother not alive | 299550 | 0.0020 | 0.0015 | 0.1857 | 0.3499 | 0.3021 | Objective socioeconomic/environmental |
| Neuroticism | Non-accidental death in close genetic family | 95591 | -0.0027 | 0.0032 | 0.3955 | 0.6252 | 0.5341 | Objective life events |
| Neuroticism | Death of a close relative | 304634 | 0.0038 | 0.0018 | 0.0373 | 0.1075 | 0.0734 | Objective life events |
| Neuroticism | Death of spouse or partner | 304634 | 0.0028 | 0.0018 | 0.1195 | 0.2703 | 0.2117 | Objective life events |
| Neuroticism | Serious illness/injury/assault of close relative | 304634 | -0.0009 | 0.0018 | 0.6200 | 0.8439 | 0.7511 | Objective life events |
| Neuroticism | Infrequent friend/family visits | 302589 | 0.0012 | 0.0018 | 0.5173 | 0.7456 | 0.6594 | Objective socioeconomic/environmental |
| Neuroticism | Loneliness/isolation | 300002 | 0.0165 | 0.0018 | 1.42e-19 | 3.48e-18 | 1.01e-18 | Perception/subjectivity |
| Neuroticism | Social isolation behaviour | 304634 | 0.0081 | 0.0018 | 6.87e-6 | 4.81e-5 | 2.23e-5 | Behavioural/lifestyle |
| Schizophrenia | Smoking | 303330 | 0.0178 | 0.0018 | 5.34e-23 | 1.31e-21 | 4.44e-22 | Behavioural/lifestyle |
| Schizophrenia | Frequent alcohol intake | 304278 | 0.0003 | 0.0018 | 0.8734 | 0.8955 | 0.9371 | Behavioural/lifestyle |
| Schizophrenia | Low moderate physical activity | 290819 | 0.0225 | 0.0019 | 9.89e-34 | 4.85e-32 | 1.04e-32 | Behavioural/lifestyle |
| Schizophrenia | Low vigorous physical activity | 290787 | 0.0139 | 0.0018 | 3.73e-14 | 4.57e-13 | 2.08e-13 | Behavioural/lifestyle |
| Schizophrenia | Low vegetable consumption | 43333 | -0.0075 | 0.0049 | 0.1214 | 0.1983 | 0.2123 | Behavioural/lifestyle |
| Schizophrenia | Low fish consumption | 43333 | -0.0007 | 0.0048 | 0.8906 | 0.8955 | 0.9441 | Behavioural/lifestyle |
| Schizophrenia | Low fruit consumption | 43333 | -0.0012 | 0.0048 | 0.8094 | 0.8955 | 0.8835 | Behavioural/lifestyle |
| Schizophrenia | Low yogurt/ice cream consumption | 43333 | -0.0006 | 0.0048 | 0.8955 | 0.8955 | 0.9448 | Behavioural/lifestyle |
| Schizophrenia | Ever taken cannabis | 83055 | 0.0241 | 0.0032 | 6.45e-14 | 6.32e-13 | 3.55e-13 | Behavioural/lifestyle |
| Schizophrenia | Marital separation or divorce | 304634 | 0.0065 | 0.0018 | 0.0003 | 0.0008 | 0.0007 | Objective life events |
| Schizophrenia | Sexual interference by partner/ex-partner without consent | 82967 | 0.0092 | 0.0033 | 0.0053 | 0.0107 | 0.0119 | Interpersonal adversity/victimisation |
| Schizophrenia | Victim of sexual assault | 82369 | 0.0219 | 0.0034 | 1.62e-10 | 1.32e-9 | 7.76e-10 | Interpersonal adversity/victimisation |
| Schizophrenia | Belittlement by partner/ex-partner | 82956 | 0.0116 | 0.0034 | 0.0007 | 0.0018 | 0.0017 | Perception/subjectivity |
| Schizophrenia | Physical violence by partner/ex-partner | 82976 | 0.0122 | 0.0035 | 0.0004 | 0.0012 | 0.0011 | Interpersonal adversity/victimisation |
| Schizophrenia | Victim of physically violent crime | 83062 | 0.0146 | 0.0034 | 1.40e-5 | 5.27e-5 | 4.34e-5 | Interpersonal adversity/victimisation |
| Schizophrenia | Felt hated by family member as a child | 83007 | 0.0146 | 0.0034 | 1.82e-5 | 6.37e-5 | 5.53e-5 | Perception/subjectivity |
| Schizophrenia | Physically abused by family as a child | 83033 | 0.0039 | 0.0035 | 0.2626 | 0.3386 | 0.3939 | Interpersonal adversity/victimisation |
| Schizophrenia | Sexually molested as a child | 82479 | 0.0144 | 0.0035 | 3.37e-5 | 0.0001 | 9.98e-5 | Interpersonal adversity/victimisation |
| Schizophrenia | Low family relationship satisfaction | 99904 | 0.0290 | 0.0032 | 6.68e-20 | 1.09e-18 | 4.83e-19 | Perception/subjectivity |
| Schizophrenia | Combat or war-zone exposure | 83097 | 0.0036 | 0.0034 | 0.2889 | 0.3538 | 0.4218 | Objective life events |
| Schizophrenia | Financial difficulties | 304634 | 0.0089 | 0.0018 | 5.99e-7 | 2.67e-6 | 2.16e-6 | Objective socioeconomic/environmental |
| Schizophrenia | Low financial situation satisfaction | 100315 | 0.0103 | 0.0032 | 0.0011 | 0.0027 | 0.0026 | Perception/subjectivity |
| Schizophrenia | Not in paid employment/self-employment | 304634 | 0.0042 | 0.0015 | 0.0044 | 0.0093 | 0.0101 | Objective socioeconomic/environmental |
| Schizophrenia | Unable to work because of sickness/disability | 304634 | 0.0056 | 0.0018 | 0.0024 | 0.0058 | 0.0057 | Objective socioeconomic/environmental |
| Schizophrenia | Unemployed | 304634 | 0.0049 | 0.0018 | 0.0073 | 0.0144 | 0.0160 | Objective socioeconomic/environmental |
| Schizophrenia | Unable to pay rent/mortgage as an adult | 81997 | 0.0079 | 0.0035 | 0.0226 | 0.0410 | 0.0470 | Objective socioeconomic/environmental |
| Schizophrenia | Non-owner accommodation / renting | 300624 | 0.0080 | 0.0017 | 1.92e-6 | 7.83e-6 | 6.71e-6 | Objective socioeconomic/environmental |
| Schizophrenia | Low average household income | 261733 | 0.0107 | 0.0018 | 1.36e-9 | 8.32e-9 | 5.99e-9 | Objective socioeconomic/environmental |
| Schizophrenia | Higher PM10 air pollution | 277926 | -0.0026 | 0.0018 | 0.1548 | 0.2299 | 0.2596 | Objective socioeconomic/environmental |
| Schizophrenia | Higher PM2.5 air pollution | 277926 | 0.0018 | 0.0018 | 0.3152 | 0.3767 | 0.4528 | Objective socioeconomic/environmental |
| Schizophrenia | Maternal smoking around birth | 261771 | 0.0058 | 0.0020 | 0.0032 | 0.0071 | 0.0076 | Behavioural/lifestyle |
| Schizophrenia | Higher nitrogen dioxide air pollution | 300348 | 0.0047 | 0.0016 | 0.0030 | 0.0070 | 0.0072 | Objective socioeconomic/environmental |
| Schizophrenia | Higher Townsend deprivation | 304310 | 0.0098 | 0.0017 | 5.97e-9 | 3.25e-8 | 2.55e-8 | Objective socioeconomic/environmental |
| Schizophrenia | Urban/rural population density | 301809 | -0.0002 | 0.0012 | 0.8936 | 0.8955 | 0.9448 | Objective socioeconomic/environmental |
| Schizophrenia | Low happiness with own health | 83016 | 0.0018 | 0.0035 | 0.6108 | 0.7125 | 0.7482 | Perception/subjectivity |
| Schizophrenia | Poor overall health rating | 303427 | 0.0100 | 0.0018 | 2.88e-8 | 1.41e-7 | 1.15e-7 | Perception/subjectivity |
| Schizophrenia | Serious life-threatening accident | 83098 | -0.0006 | 0.0034 | 0.8628 | 0.8955 | 0.9326 | Objective life events |
| Schizophrenia | Diagnosed with life-threatening illness | 82917 | 0.0066 | 0.0034 | 0.0525 | 0.0888 | 0.1007 | Objective life events |
| Schizophrenia | Serious illness/injury/assault to self | 304634 | 0.0047 | 0.0018 | 0.0096 | 0.0182 | 0.0208 | Objective life events |
| Schizophrenia | Poor health sum score | 304634 | -0.0023 | 0.0018 | 0.1854 | 0.2672 | 0.3021 | Objective socioeconomic/environmental |
| Schizophrenia | Father not alive | 296617 | -0.0003 | 0.0016 | 0.8691 | 0.8955 | 0.9361 | Objective socioeconomic/environmental |
| Schizophrenia | Mother not alive | 299550 | -0.0019 | 0.0016 | 0.2286 | 0.3135 | 0.3525 | Objective socioeconomic/environmental |
| Schizophrenia | Non-accidental death in close genetic family | 95591 | 0.0046 | 0.0032 | 0.1539 | 0.2299 | 0.2590 | Objective life events |
| Schizophrenia | Death of a close relative | 304634 | 0.0020 | 0.0018 | 0.2799 | 0.3516 | 0.4116 | Objective life events |
| Schizophrenia | Death of spouse or partner | 304634 | 0.0037 | 0.0018 | 0.0364 | 0.0637 | 0.0720 | Objective life events |
| Schizophrenia | Serious illness/injury/assault of close relative | 304634 | -0.0022 | 0.0018 | 0.2367 | 0.3135 | 0.3600 | Objective life events |
| Schizophrenia | Infrequent friend/family visits | 302589 | 0.0021 | 0.0018 | 0.2361 | 0.3135 | 0.3600 | Objective socioeconomic/environmental |
| Schizophrenia | Loneliness/isolation | 300002 | 0.0115 | 0.0018 | 3.39e-10 | 2.38e-9 | 1.58e-9 | Perception/subjectivity |
| Schizophrenia | Social isolation behaviour | 304634 | 0.0027 | 0.0018 | 0.1305 | 0.2063 | 0.2231 | Behavioural/lifestyle |

# Supplementary Table 4A. Final five-category pooled estimates

| **PRS** | **Category** | **No. ERFs** | **beta** | **se** | **95% CI lower** | **95% CI upper** | **p** |
| --- | --- | --- | --- | --- | --- | --- | --- |
| Schizophrenia | Perception / subjectivity | 7 | 0.0121 | 0.0010 | 0.0102 | 0.0140 | 1.26e-35 |
| Schizophrenia | Interpersonal adversity / victimisation | 6 | 0.0127 | 0.0014 | 0.0100 | 0.0154 | 7.95e-20 |
| Schizophrenia | Objective life events | 9 | 0.0031 | 0.0007 | 0.0017 | 0.0045 | 2.05e-5 |
| Schizophrenia | Objective socioeconomic / environmental | 16 | 0.0033 | 0.0004 | 0.0025 | 0.0041 | 2.64e-15 |
| Schizophrenia | Behavioural / lifestyle | 11 | 0.0100 | 0.0007 | 0.0086 | 0.0114 | 1.94e-46 |
| Bipolar disorder | Perception / subjectivity | 7 | 0.0080 | 0.0010 | 0.0061 | 0.0099 | 1.99e-16 |
| Bipolar disorder | Interpersonal adversity / victimisation | 6 | 0.0110 | 0.0014 | 0.0083 | 0.0137 | 3.51e-15 |
| Bipolar disorder | Objective life events | 9 | 0.0039 | 0.0007 | 0.0024 | 0.0053 | 1.39e-7 |
| Bipolar disorder | Objective socioeconomic / environmental | 16 | 0.0009 | 0.0004 | 8.98e-5 | 0.0017 | 0.0298 |
| Bipolar disorder | Behavioural / lifestyle | 11 | 0.0071 | 0.0007 | 0.0057 | 0.0084 | 9.49e-24 |
| Major depression | Perception / subjectivity | 7 | 0.0435 | 0.0010 | 0.0416 | 0.0454 | <1e-300 |
| Major depression | Interpersonal adversity / victimisation | 6 | 0.0217 | 0.0014 | 0.0189 | 0.0244 | 1.50e-53 |
| Major depression | Objective life events | 9 | 0.0096 | 0.0007 | 0.0082 | 0.0110 | 2.53e-39 |
| Major depression | Objective socioeconomic / environmental | 16 | 0.0125 | 0.0004 | 0.0117 | 0.0133 | 9.77e-195 |
| Major depression | Behavioural / lifestyle | 11 | 0.0163 | 0.0007 | 0.0149 | 0.0177 | 3.90e-120 |
| ADHD | Perception / subjectivity | 7 | 0.0319 | 0.0010 | 0.0300 | 0.0338 | 1.09e-238 |
| ADHD | Interpersonal adversity / victimisation | 6 | 0.0227 | 0.0014 | 0.0199 | 0.0255 | 1.40e-58 |
| ADHD | Objective life events | 9 | 0.0070 | 0.0007 | 0.0055 | 0.0084 | 1.55e-21 |
| ADHD | Objective socioeconomic / environmental | 16 | 0.0136 | 0.0004 | 0.0128 | 0.0144 | 3.79e-230 |
| ADHD | Behavioural / lifestyle | 11 | 0.0182 | 0.0007 | 0.0168 | 0.0196 | 6.58e-150 |
| Educational attainment | Perception / subjectivity | 7 | -0.0290 | 0.0010 | -0.0310 | -0.0271 | 4.51e-194 |
| Educational attainment | Interpersonal adversity / victimisation | 6 | -0.0025 | 0.0014 | -0.0052 | 0.0003 | 0.0777 |
| Educational attainment | Objective life events | 9 | -0.0026 | 0.0007 | -0.0041 | -0.0012 | 0.0003 |
| Educational attainment | Objective socioeconomic / environmental | 16 | -0.0184 | 0.0004 | -0.0192 | -0.0175 | <1e-300 |
| Educational attainment | Behavioural / lifestyle | 11 | -0.0132 | 0.0007 | -0.0146 | -0.0118 | 1.71e-78 |
| Neuroticism | Perception / subjectivity | 7 | 0.0145 | 0.0010 | 0.0126 | 0.0164 | 1.59e-50 |
| Neuroticism | Interpersonal adversity / victimisation | 6 | 0.0027 | 0.0014 | -0.0001 | 0.0054 | 0.0616 |
| Neuroticism | Objective life events | 9 | 0.0014 | 0.0007 | -1.20e-5 | 0.0028 | 0.0520 |
| Neuroticism | Objective socioeconomic / environmental | 16 | 0.0020 | 0.0004 | 0.0012 | 0.0028 | 1.31e-6 |
| Neuroticism | Behavioural / lifestyle | 11 | 0.0052 | 0.0007 | 0.0038 | 0.0066 | 8.34e-14 |
| Extraversion | Perception / subjectivity | 7 | -0.0041 | 0.0010 | -0.0060 | -0.0022 | 2.57e-5 |
| Extraversion | Interpersonal adversity / victimisation | 6 | -0.0007 | 0.0014 | -0.0034 | 0.0021 | 0.6357 |
| Extraversion | Objective life events | 9 | 0.0021 | 0.0007 | 0.0007 | 0.0036 | 0.0036 |
| Extraversion | Objective socioeconomic / environmental | 16 | 0.0006 | 0.0004 | -0.0003 | 0.0014 | 0.1778 |
| Extraversion | Behavioural / lifestyle | 11 | 0.0003 | 0.0007 | -0.0010 | 0.0017 | 0.6304 |
| Alzheimer's disease | Perception / subjectivity | 7 | 0.0012 | 0.0010 | -0.0007 | 0.0031 | 0.2127 |
| Alzheimer's disease | Interpersonal adversity / victimisation | 6 | -0.0012 | 0.0014 | -0.0039 | 0.0016 | 0.4062 |
| Alzheimer's disease | Objective life events | 9 | -0.0004 | 0.0007 | -0.0018 | 0.0010 | 0.5887 |
| Alzheimer's disease | Objective socioeconomic / environmental | 16 | 0.0003 | 0.0004 | -0.0006 | 0.0011 | 0.5356 |
| Alzheimer's disease | Behavioural / lifestyle | 11 | 0.0019 | 0.0007 | 0.0005 | 0.0032 | 0.0071 |
| LDL cholesterol | Perception / subjectivity | 7 | 0.0048 | 0.0010 | 0.0029 | 0.0067 | 6.04e-7 |
| LDL cholesterol | Interpersonal adversity / victimisation | 6 | 0.0012 | 0.0014 | -0.0015 | 0.0040 | 0.3845 |
| LDL cholesterol | Objective life events | 9 | 0.0013 | 0.0007 | -8.08e-5 | 0.0028 | 0.0645 |
| LDL cholesterol | Objective socioeconomic / environmental | 16 | 0.0025 | 0.0004 | 0.0017 | 0.0033 | 2.23e-9 |
| LDL cholesterol | Behavioural / lifestyle | 11 | 0.0006 | 0.0007 | -0.0007 | 0.0020 | 0.3733 |

# Supplementary Table 4B. Pairwise contrasts between final ERF categories

| **PRS** | **group1** | **group2** | **beta_difference** | **se_difference** | **z** | **p_difference** |
| --- | --- | --- | --- | --- | --- | --- |
| Schizophrenia | Perception / subjectivity | Interpersonal adversity / victimisation | -0.0006 | 0.0017 | -0.3531 | 0.7240 |
| Schizophrenia | Perception / subjectivity | Objective life events | 0.0090 | 0.0012 | 7.4166 | 1.20e-13 |
| Schizophrenia | Perception / subjectivity | Objective socioeconomic / environmental | 0.0088 | 0.0011 | 8.3007 | 1.04e-16 |
| Schizophrenia | Perception / subjectivity | Behavioural / lifestyle | 0.0021 | 0.0012 | 1.7451 | 0.0810 |
| Schizophrenia | Interpersonal adversity / victimisation | Objective life events | 0.0096 | 0.0016 | 6.1071 | 1.01e-9 |
| Schizophrenia | Interpersonal adversity / victimisation | Objective socioeconomic / environmental | 0.0094 | 0.0015 | 6.4469 | 1.14e-10 |
| Schizophrenia | Interpersonal adversity / victimisation | Behavioural / lifestyle | 0.0027 | 0.0016 | 1.7243 | 0.0846 |
| Schizophrenia | Objective life events | Objective socioeconomic / environmental | -0.0002 | 0.0008 | -0.2599 | 0.7950 |
| Schizophrenia | Objective life events | Behavioural / lifestyle | -0.0069 | 0.0010 | -6.8470 | 7.54e-12 |
| Schizophrenia | Objective socioeconomic / environmental | Behavioural / lifestyle | -0.0067 | 0.0008 | -8.2041 | 2.32e-16 |
| Bipolar disorder | Perception / subjectivity | Interpersonal adversity / victimisation | -0.0030 | 0.0017 | -1.7448 | 0.0810 |
| Bipolar disorder | Perception / subjectivity | Objective life events | 0.0042 | 0.0012 | 3.4125 | 0.0006 |
| Bipolar disorder | Perception / subjectivity | Objective socioeconomic / environmental | 0.0071 | 0.0011 | 6.6848 | 2.31e-11 |
| Bipolar disorder | Perception / subjectivity | Behavioural / lifestyle | 0.0010 | 0.0012 | 0.7982 | 0.4247 |
| Bipolar disorder | Interpersonal adversity / victimisation | Objective life events | 0.0071 | 0.0016 | 4.5243 | 6.06e-6 |
| Bipolar disorder | Interpersonal adversity / victimisation | Objective socioeconomic / environmental | 0.0101 | 0.0015 | 6.9069 | 4.95e-12 |
| Bipolar disorder | Interpersonal adversity / victimisation | Behavioural / lifestyle | 0.0039 | 0.0016 | 2.5149 | 0.0119 |
| Bipolar disorder | Objective life events | Objective socioeconomic / environmental | 0.0029 | 0.0008 | 3.4796 | 0.0005 |
| Bipolar disorder | Objective life events | Behavioural / lifestyle | -0.0032 | 0.0010 | -3.1553 | 0.0016 |
| Bipolar disorder | Objective socioeconomic / environmental | Behavioural / lifestyle | -0.0061 | 0.0008 | -7.4963 | 6.57e-14 |
| Major depression | Perception / subjectivity | Interpersonal adversity / victimisation | 0.0218 | 0.0017 | 12.7544 | 2.95e-37 |
| Major depression | Perception / subjectivity | Objective life events | 0.0339 | 0.0012 | 27.8519 | 1.02e-170 |
| Major depression | Perception / subjectivity | Objective socioeconomic / environmental | 0.0310 | 0.0011 | 29.2656 | 2.84e-188 |
| Major depression | Perception / subjectivity | Behavioural / lifestyle | 0.0272 | 0.0012 | 22.6822 | 6.71e-114 |
| Major depression | Interpersonal adversity / victimisation | Objective life events | 0.0121 | 0.0016 | 7.6139 | 2.66e-14 |
| Major depression | Interpersonal adversity / victimisation | Objective socioeconomic / environmental | 0.0092 | 0.0015 | 6.2491 | 4.13e-10 |
| Major depression | Interpersonal adversity / victimisation | Behavioural / lifestyle | 0.0054 | 0.0016 | 3.4103 | 0.0006 |
| Major depression | Objective life events | Objective socioeconomic / environmental | -0.0029 | 0.0008 | -3.4366 | 0.0006 |
| Major depression | Objective life events | Behavioural / lifestyle | -0.0067 | 0.0010 | -6.6311 | 3.33e-11 |
| Major depression | Objective socioeconomic / environmental | Behavioural / lifestyle | -0.0038 | 0.0008 | -4.6745 | 2.95e-6 |
| ADHD | Perception / subjectivity | Interpersonal adversity / victimisation | 0.0092 | 0.0017 | 5.3906 | 7.02e-8 |
| ADHD | Perception / subjectivity | Objective life events | 0.0250 | 0.0012 | 20.6033 | 2.57e-94 |
| ADHD | Perception / subjectivity | Objective socioeconomic / environmental | 0.0183 | 0.0011 | 17.3951 | 8.98e-68 |
| ADHD | Perception / subjectivity | Behavioural / lifestyle | 0.0137 | 0.0012 | 11.5111 | 1.16e-30 |
| ADHD | Interpersonal adversity / victimisation | Objective life events | 0.0158 | 0.0016 | 9.9406 | 2.77e-23 |
| ADHD | Interpersonal adversity / victimisation | Objective socioeconomic / environmental | 0.0091 | 0.0015 | 6.2204 | 4.96e-10 |
| ADHD | Interpersonal adversity / victimisation | Behavioural / lifestyle | 0.0045 | 0.0016 | 2.8793 | 0.0040 |
| ADHD | Objective life events | Objective socioeconomic / environmental | -0.0066 | 0.0008 | -7.8726 | 3.47e-15 |
| ADHD | Objective life events | Behavioural / lifestyle | -0.0112 | 0.0010 | -11.1320 | 8.76e-29 |
| ADHD | Objective socioeconomic / environmental | Behavioural / lifestyle | -0.0046 | 0.0008 | -5.6674 | 1.45e-8 |
| Educational attainment | Perception / subjectivity | Interpersonal adversity / victimisation | -0.0266 | 0.0017 | -15.5013 | 3.40e-54 |
| Educational attainment | Perception / subjectivity | Objective life events | -0.0264 | 0.0012 | -21.5675 | 3.63e-103 |
| Educational attainment | Perception / subjectivity | Objective socioeconomic / environmental | -0.0107 | 0.0011 | -10.0183 | 1.27e-23 |
| Educational attainment | Perception / subjectivity | Behavioural / lifestyle | -0.0158 | 0.0012 | -13.1570 | 1.55e-39 |
| Educational attainment | Interpersonal adversity / victimisation | Objective life events | 0.0002 | 0.0016 | 0.1048 | 0.9165 |
| Educational attainment | Interpersonal adversity / victimisation | Objective socioeconomic / environmental | 0.0159 | 0.0015 | 10.8101 | 3.08e-27 |
| Educational attainment | Interpersonal adversity / victimisation | Behavioural / lifestyle | 0.0107 | 0.0016 | 6.8102 | 9.75e-12 |
| Educational attainment | Objective life events | Objective socioeconomic / environmental | 0.0157 | 0.0008 | 18.5016 | 2.00e-76 |
| Educational attainment | Objective life events | Behavioural / lifestyle | 0.0105 | 0.0010 | 10.3559 | 3.94e-25 |
| Educational attainment | Objective socioeconomic / environmental | Behavioural / lifestyle | -0.0052 | 0.0008 | -6.2974 | 3.03e-10 |
| Neuroticism | Perception / subjectivity | Interpersonal adversity / victimisation | 0.0119 | 0.0017 | 6.8973 | 5.30e-12 |
| Neuroticism | Perception / subjectivity | Objective life events | 0.0131 | 0.0012 | 10.7857 | 4.02e-27 |
| Neuroticism | Perception / subjectivity | Objective socioeconomic / environmental | 0.0125 | 0.0011 | 11.8126 | 3.36e-32 |
| Neuroticism | Perception / subjectivity | Behavioural / lifestyle | 0.0093 | 0.0012 | 7.7715 | 7.76e-15 |
| Neuroticism | Interpersonal adversity / victimisation | Objective life events | 0.0012 | 0.0016 | 0.7744 | 0.4387 |
| Neuroticism | Interpersonal adversity / victimisation | Objective socioeconomic / environmental | 0.0006 | 0.0015 | 0.4246 | 0.6711 |
| Neuroticism | Interpersonal adversity / victimisation | Behavioural / lifestyle | -0.0026 | 0.0016 | -1.6207 | 0.1051 |
| Neuroticism | Objective life events | Objective socioeconomic / environmental | -0.0006 | 0.0008 | -0.7224 | 0.4700 |
| Neuroticism | Objective life events | Behavioural / lifestyle | -0.0038 | 0.0010 | -3.7620 | 0.0002 |
| Neuroticism | Objective socioeconomic / environmental | Behavioural / lifestyle | -0.0032 | 0.0008 | -3.9189 | 8.90e-5 |
| Extraversion | Perception / subjectivity | Interpersonal adversity / victimisation | -0.0034 | 0.0017 | -2.0023 | 0.0453 |
| Extraversion | Perception / subjectivity | Objective life events | -0.0062 | 0.0012 | -5.1112 | 3.20e-7 |
| Extraversion | Perception / subjectivity | Objective socioeconomic / environmental | -0.0047 | 0.0011 | -4.3977 | 1.09e-5 |
| Extraversion | Perception / subjectivity | Behavioural / lifestyle | -0.0044 | 0.0012 | -3.6962 | 0.0002 |
| Extraversion | Interpersonal adversity / victimisation | Objective life events | -0.0028 | 0.0016 | -1.7650 | 0.0776 |
| Extraversion | Interpersonal adversity / victimisation | Objective socioeconomic / environmental | -0.0012 | 0.0015 | -0.8389 | 0.4015 |
| Extraversion | Interpersonal adversity / victimisation | Behavioural / lifestyle | -0.0010 | 0.0016 | -0.6385 | 0.5232 |
| Extraversion | Objective life events | Objective socioeconomic / environmental | 0.0016 | 0.0008 | 1.8566 | 0.0634 |
| Extraversion | Objective life events | Behavioural / lifestyle | 0.0018 | 0.0010 | 1.7726 | 0.0763 |
| Extraversion | Objective socioeconomic / environmental | Behavioural / lifestyle | 0.0002 | 0.0008 | 0.2799 | 0.7796 |
| Alzheimer's disease | Perception / subjectivity | Interpersonal adversity / victimisation | 0.0024 | 0.0017 | 1.3900 | 0.1645 |
| Alzheimer's disease | Perception / subjectivity | Objective life events | 0.0016 | 0.0012 | 1.3208 | 0.1866 |
| Alzheimer's disease | Perception / subjectivity | Objective socioeconomic / environmental | 0.0009 | 0.0011 | 0.8982 | 0.3691 |
| Alzheimer's disease | Perception / subjectivity | Behavioural / lifestyle | -0.0007 | 0.0012 | -0.5609 | 0.5749 |
| Alzheimer's disease | Interpersonal adversity / victimisation | Objective life events | -0.0008 | 0.0016 | -0.4898 | 0.6243 |
| Alzheimer's disease | Interpersonal adversity / victimisation | Objective socioeconomic / environmental | -0.0014 | 0.0015 | -0.9725 | 0.3308 |
| Alzheimer's disease | Interpersonal adversity / victimisation | Behavioural / lifestyle | -0.0030 | 0.0016 | -1.9371 | 0.0527 |
| Alzheimer's disease | Objective life events | Objective socioeconomic / environmental | -0.0007 | 0.0008 | -0.7774 | 0.4369 |
| Alzheimer's disease | Objective life events | Behavioural / lifestyle | -0.0023 | 0.0010 | -2.2517 | 0.0243 |
| Alzheimer's disease | Objective socioeconomic / environmental | Behavioural / lifestyle | -0.0016 | 0.0008 | -1.9895 | 0.0466 |
| LDL cholesterol | Perception / subjectivity | Interpersonal adversity / victimisation | 0.0036 | 0.0017 | 2.1138 | 0.0345 |
| LDL cholesterol | Perception / subjectivity | Objective life events | 0.0035 | 0.0012 | 2.8813 | 0.0040 |
| LDL cholesterol | Perception / subjectivity | Objective socioeconomic / environmental | 0.0023 | 0.0011 | 2.2122 | 0.0270 |
| LDL cholesterol | Perception / subjectivity | Behavioural / lifestyle | 0.0042 | 0.0012 | 3.5316 | 0.0004 |
| LDL cholesterol | Interpersonal adversity / victimisation | Objective life events | -0.0001 | 0.0016 | -0.0762 | 0.9392 |
| LDL cholesterol | Interpersonal adversity / victimisation | Objective socioeconomic / environmental | -0.0013 | 0.0015 | -0.8698 | 0.3844 |
| LDL cholesterol | Interpersonal adversity / victimisation | Behavioural / lifestyle | 0.0006 | 0.0016 | 0.3843 | 0.7007 |
| LDL cholesterol | Objective life events | Objective socioeconomic / environmental | -0.0012 | 0.0008 | -1.3781 | 0.1682 |
| LDL cholesterol | Objective life events | Behavioural / lifestyle | 0.0007 | 0.0010 | 0.7191 | 0.4721 |
| LDL cholesterol | Objective socioeconomic / environmental | Behavioural / lifestyle | 0.0019 | 0.0008 | 2.3138 | 0.0207 |

# Supplementary Table 5A. Broad 7-versus-25 perception/subjectivity comparison

| **PRS** | **Group** | **No. ERFs** | **beta** | **se** | **95% CI lower** | **95% CI upper** | **p** | **Difference P** |
| --- | --- | --- | --- | --- | --- | --- | --- | --- |
| Schizophrenia | Perception / subjectivity | 7 | 0.0121 | 0.0010 | 0.0102 | 0.0140 | 1.26e-35 | 1.58e-17 |
| Schizophrenia | Objective / non-perception | 25 | 0.0033 | 0.0004 | 0.0025 | 0.0040 | 2.77e-19 | 1.58e-17 |
| Bipolar disorder | Perception / subjectivity | 7 | 0.0080 | 0.0010 | 0.0061 | 0.0099 | 1.99e-16 | 9.54e-10 |
| Bipolar disorder | Objective / non-perception | 25 | 0.0016 | 0.0004 | 0.0009 | 0.0024 | 6.46e-6 | 9.54e-10 |
| Major depression | Perception / subjectivity | 7 | 0.0435 | 0.0010 | 0.0416 | 0.0454 | <1e-300 | 6.62e-205 |
| Major depression | Objective / non-perception | 25 | 0.0118 | 0.0004 | 0.0111 | 0.0125 | 1.38e-229 | 6.62e-205 |
| ADHD | Perception / subjectivity | 7 | 0.0319 | 0.0010 | 0.0300 | 0.0338 | 1.09e-238 | 2.60e-83 |
| ADHD | Objective / non-perception | 25 | 0.0119 | 0.0004 | 0.0112 | 0.0126 | 2.01e-236 | 2.60e-83 |
| Educational attainment | Perception / subjectivity | 7 | -0.0290 | 0.0010 | -0.0310 | -0.0271 | 4.51e-194 | 2.49e-44 |
| Educational attainment | Objective / non-perception | 25 | -0.0145 | 0.0004 | -0.0152 | -0.0137 | <1e-300 | 2.49e-44 |
| Neuroticism | Perception / subjectivity | 7 | 0.0145 | 0.0010 | 0.0126 | 0.0164 | 1.59e-50 | 3.35e-34 |
| Neuroticism | Objective / non-perception | 25 | 0.0019 | 0.0004 | 0.0012 | 0.0026 | 2.43e-7 | 3.35e-34 |
| Extraversion | Perception / subjectivity | 7 | -0.0041 | 0.0010 | -0.0060 | -0.0022 | 2.57e-5 | 1.19e-6 |
| Extraversion | Objective / non-perception | 25 | 0.0010 | 0.0004 | 0.0002 | 0.0017 | 0.0090 | 1.19e-6 |
| Alzheimer's disease | Perception / subjectivity | 7 | 0.0012 | 0.0010 | -0.0007 | 0.0031 | 0.2127 | 0.2833 |
| Alzheimer's disease | Objective / non-perception | 25 | 9.69e-5 | 0.0004 | -0.0006 | 0.0008 | 0.7888 | 0.2833 |
| LDL cholesterol | Perception / subjectivity | 7 | 0.0048 | 0.0010 | 0.0029 | 0.0067 | 6.04e-7 | 0.0113 |
| LDL cholesterol | Objective / non-perception | 25 | 0.0022 | 0.0004 | 0.0015 | 0.0029 | 1.02e-9 | 0.0113 |

# Supplementary Table 5B. Primary matched four-pair pooled comparison

| **PRS** | **Group** | **No. ERFs** | **beta** | **se** | **95% CI lower** | **95% CI upper** | **p** | **Difference P / FDR q** |
| --- | --- | --- | --- | --- | --- | --- | --- | --- |
| Schizophrenia | Perception / subjectivity | 4 | 0.0129 | 0.0013 | 0.0103 | 0.0154 | 8.31e-23 | P=2.83e-6; q=8.50e-6 |
| Schizophrenia | Objective / non-perception | 4 | 0.0052 | 0.0010 | 0.0032 | 0.0071 | 2.01e-7 | P=2.83e-6; q=8.50e-6 |
| Bipolar disorder | Perception / subjectivity | 4 | 0.0100 | 0.0013 | 0.0074 | 0.0125 | 3.61e-14 | P=1.33e-11; q=6.00e-11 |
| Bipolar disorder | Objective / non-perception | 4 | -0.0012 | 0.0010 | -0.0032 | 0.0008 | 0.2282 | P=1.33e-11; q=6.00e-11 |
| Major depression | Perception / subjectivity | 4 | 0.0417 | 0.0013 | 0.0391 | 0.0442 | 1.65e-222 | P=6.63e-29; q=5.97e-28 |
| Major depression | Objective / non-perception | 4 | 0.0233 | 0.0010 | 0.0214 | 0.0253 | 1.46e-121 | P=6.63e-29; q=5.97e-28 |
| ADHD | Perception / subjectivity | 4 | 0.0245 | 0.0013 | 0.0219 | 0.0270 | 2.97e-78 | P=7.63e-6; q=1.72e-5 |
| ADHD | Objective / non-perception | 4 | 0.0171 | 0.0010 | 0.0152 | 0.0191 | 2.92e-66 | P=7.63e-6; q=1.72e-5 |
| Educational attainment | Perception / subjectivity | 4 | -0.0176 | 0.0013 | -0.0201 | -0.0150 | 1.10e-40 | P=0.0083; q=0.0124 |
| Educational attainment | Objective / non-perception | 4 | -0.0219 | 0.0010 | -0.0239 | -0.0200 | 1.93e-106 | P=0.0083; q=0.0124 |
| Neuroticism | Perception / subjectivity | 4 | 0.0142 | 0.0013 | 0.0116 | 0.0168 | 1.28e-27 | P=8.84e-5; q=0.0002 |
| Neuroticism | Objective / non-perception | 4 | 0.0078 | 0.0010 | 0.0058 | 0.0097 | 4.76e-15 | P=8.84e-5; q=0.0002 |
| Extraversion | Perception / subjectivity | 4 | -0.0061 | 0.0013 | -0.0087 | -0.0035 | 3.06e-6 | P=0.0498; q=0.0560 |
| Extraversion | Objective / non-perception | 4 | -0.0093 | 0.0010 | -0.0113 | -0.0074 | 5.85e-21 | P=0.0498; q=0.0560 |
| Alzheimer's disease | Perception / subjectivity | 4 | 0.0018 | 0.0013 | -0.0007 | 0.0044 | 0.1572 | P=0.0232; q=0.0298 |
| Alzheimer's disease | Objective / non-perception | 4 | -0.0019 | 0.0010 | -0.0038 | 6.60e-5 | 0.0584 | P=0.0232; q=0.0298 |
| LDL cholesterol | Perception / subjectivity | 4 | 0.0020 | 0.0013 | -0.0005 | 0.0046 | 0.1157 | P=0.2461; q=0.2461 |
| LDL cholesterol | Objective / non-perception | 4 | 0.0039 | 0.0010 | 0.0020 | 0.0059 | 6.84e-5 | P=0.2461; q=0.2461 |

# Supplementary Table 5C. Individual results for the four primary matched pairs

| **PRS** | **pair** | **perception_risk_label** | **objective_risk_label** | **perception_beta** | **perception_se** | **objective_beta** | **objective_se** | **beta_difference** | **se_difference** | **p_difference / FDR q** |
| --- | --- | --- | --- | --- | --- | --- | --- | --- | --- | --- |
| Schizophrenia | Social disconnection | Loneliness/isolation | Social isolation behaviour | 0.0115 | 0.0018 | 0.0027 | 0.0018 | 0.0087 | 0.0026 | P=0.0007; q=0.0016 |
| Schizophrenia | Health | Low happiness with own health | Poor overall health rating | 0.0018 | 0.0035 | 0.0100 | 0.0018 | -0.0083 | 0.0039 | P=0.0351; q=0.0703 |
| Schizophrenia | Financial circumstances | Low financial situation satisfaction | Able to pay rent mortgage as an adult | 0.0103 | 0.0032 | 0.0079 | 0.0035 | 0.0024 | 0.0047 | P=0.6132; q=0.6556 |
| Schizophrenia | Family relationships | Low family relationship satisfaction | Frequency of friend family visits | 0.0290 | 0.0032 | 0.0021 | 0.0018 | 0.0269 | 0.0036 | P=1.50e-13; q=1.08e-12 |
| Bipolar disorder | Social disconnection | Loneliness/isolation | Social isolation behaviour | 0.0091 | 0.0018 | -0.0057 | 0.0018 | 0.0147 | 0.0026 | P=1.04e-8; q=4.70e-8 |
| Bipolar disorder | Health | Low happiness with own health | Poor overall health rating | 0.0003 | 0.0035 | 0.0030 | 0.0018 | -0.0027 | 0.0039 | P=0.4961; q=0.5762 |
| Bipolar disorder | Financial circumstances | Low financial situation satisfaction | Able to pay rent mortgage as an adult | 0.0037 | 0.0032 | 0.0013 | 0.0035 | 0.0023 | 0.0047 | P=0.6192; q=0.6556 |
| Bipolar disorder | Family relationships | Low family relationship satisfaction | Frequency of friend family visits | 0.0269 | 0.0032 | -0.0016 | 0.0018 | 0.0285 | 0.0036 | P=5.46e-15; q=4.91e-14 |
| Major depression | Social disconnection | Loneliness/isolation | Social isolation behaviour | 0.0440 | 0.0018 | 0.0196 | 0.0018 | 0.0244 | 0.0026 | P=1.76e-21; q=2.11e-20 |
| Major depression | Health | Low happiness with own health | Poor overall health rating | 0.0325 | 0.0035 | 0.0523 | 0.0018 | -0.0198 | 0.0039 | P=5.10e-7; q=1.84e-6 |
| Major depression | Financial circumstances | Low financial situation satisfaction | Able to pay rent mortgage as an adult | 0.0433 | 0.0031 | 0.0187 | 0.0035 | 0.0245 | 0.0047 | P=1.64e-7; q=6.57e-7 |
| Major depression | Family relationships | Low family relationship satisfaction | Frequency of friend family visits | 0.0404 | 0.0032 | -2.87e-5 | 0.0018 | 0.0404 | 0.0036 | P=1.20e-28; q=2.17e-27 |
| ADHD | Social disconnection | Loneliness/isolation | Social isolation behaviour | 0.0243 | 0.0018 | 0.0150 | 0.0018 | 0.0093 | 0.0026 | P=0.0003; q=0.0008 |
| ADHD | Health | Low happiness with own health | Poor overall health rating | 0.0217 | 0.0035 | 0.0480 | 0.0018 | -0.0263 | 0.0039 | P=2.06e-11; q=1.24e-10 |
| ADHD | Financial circumstances | Low financial situation satisfaction | Able to pay rent mortgage as an adult | 0.0394 | 0.0031 | 0.0189 | 0.0035 | 0.0205 | 0.0047 | P=1.31e-5; q=4.30e-5 |
| ADHD | Family relationships | Low family relationship satisfaction | Frequency of friend family visits | 0.0117 | 0.0032 | -0.0118 | 0.0018 | 0.0235 | 0.0036 | P=8.94e-11; q=4.60e-10 |
| Educational attainment | Social disconnection | Loneliness/isolation | Social isolation behaviour | -0.0230 | 0.0018 | -0.0195 | 0.0018 | -0.0034 | 0.0026 | P=0.1813; q=0.2507 |
| Educational attainment | Health | Low happiness with own health | Poor overall health rating | -0.0142 | 0.0035 | -0.0676 | 0.0018 | 0.0534 | 0.0040 | P=2.23e-41; q=8.02e-40 |
| Educational attainment | Financial circumstances | Low financial situation satisfaction | Able to pay rent mortgage as an adult | -0.0401 | 0.0031 | -0.0417 | 0.0035 | 0.0016 | 0.0047 | P=0.7357; q=0.7357 |
| Educational attainment | Family relationships | Low family relationship satisfaction | Frequency of friend family visits | 0.0186 | 0.0032 | 0.0249 | 0.0018 | -0.0063 | 0.0036 | P=0.0823; q=0.1559 |
| Neuroticism | Social disconnection | Loneliness/isolation | Social isolation behaviour | 0.0165 | 0.0018 | 0.0081 | 0.0018 | 0.0084 | 0.0026 | P=0.0011; q=0.0025 |
| Neuroticism | Health | Low happiness with own health | Poor overall health rating | 0.0114 | 0.0035 | 0.0166 | 0.0018 | -0.0052 | 0.0039 | P=0.1823; q=0.2507 |
| Neuroticism | Financial circumstances | Low financial situation satisfaction | Able to pay rent mortgage as an adult | 0.0098 | 0.0031 | -0.0009 | 0.0035 | 0.0107 | 0.0047 | P=0.0220; q=0.0467 |
| Neuroticism | Family relationships | Low family relationship satisfaction | Frequency of friend family visits | 0.0142 | 0.0032 | 0.0012 | 0.0018 | 0.0131 | 0.0036 | P=0.0003; q=0.0008 |
| Extraversion | Social disconnection | Loneliness/isolation | Social isolation behaviour | -0.0070 | 0.0018 | -0.0174 | 0.0018 | 0.0104 | 0.0026 | P=4.88e-5; q=0.0001 |
| Extraversion | Health | Low happiness with own health | Poor overall health rating | -0.0014 | 0.0035 | -3.75e-5 | 0.0018 | -0.0014 | 0.0039 | P=0.7233; q=0.7357 |
| Extraversion | Financial circumstances | Low financial situation satisfaction | Able to pay rent mortgage as an adult | -0.0016 | 0.0031 | 0.0046 | 0.0035 | -0.0062 | 0.0047 | P=0.1880; q=0.2507 |
| Extraversion | Family relationships | Low family relationship satisfaction | Frequency of friend family visits | -0.0121 | 0.0032 | -0.0142 | 0.0018 | 0.0021 | 0.0036 | P=0.5642; q=0.6347 |
| Alzheimer's disease | Social disconnection | Loneliness/isolation | Social isolation behaviour | 0.0013 | 0.0018 | -0.0019 | 0.0018 | 0.0031 | 0.0026 | P=0.2228; q=0.2864 |
| Alzheimer's disease | Health | Low happiness with own health | Poor overall health rating | 0.0022 | 0.0035 | -0.0014 | 0.0018 | 0.0036 | 0.0039 | P=0.3575; q=0.4291 |
| Alzheimer's disease | Financial circumstances | Low financial situation satisfaction | Able to pay rent mortgage as an adult | 0.0018 | 0.0031 | -0.0048 | 0.0034 | 0.0065 | 0.0046 | P=0.1560; q=0.2442 |
| Alzheimer's disease | Family relationships | Low family relationship satisfaction | Frequency of friend family visits | 0.0034 | 0.0031 | -0.0015 | 0.0018 | 0.0049 | 0.0036 | P=0.1710; q=0.2507 |
| LDL cholesterol | Social disconnection | Loneliness/isolation | Social isolation behaviour | -0.0013 | 0.0018 | 0.0011 | 0.0018 | -0.0025 | 0.0026 | P=0.3284; q=0.4076 |
| LDL cholesterol | Health | Low happiness with own health | Poor overall health rating | 0.0076 | 0.0035 | 0.0143 | 0.0018 | -0.0066 | 0.0039 | P=0.0923; q=0.1662 |
| LDL cholesterol | Financial circumstances | Low financial situation satisfaction | Able to pay rent mortgage as an adult | 0.0064 | 0.0031 | -0.0010 | 0.0035 | 0.0075 | 0.0047 | P=0.1102; q=0.1890 |
| LDL cholesterol | Family relationships | Low family relationship satisfaction | Frequency of friend family visits | 0.0032 | 0.0031 | -0.0021 | 0.0018 | 0.0053 | 0.0036 | P=0.1416; q=0.2316 |

# Analysis note

All 441 primary models adjusted for age, sex, the first 15 genetic principal components, genotyping batch and assessment centre. Batch and assessment centre were entered as categorical factors. Analyses used model-specific complete cases; N ranged from 43,333 to 304,634. Within-PRS FDR correction was applied separately across the 49 environmental risk factors for each PRS. The broad comparison included seven perception/subjectivity ERFs and 25 objective/non-perception ERFs and excluded all six interpersonal adversity/victimisation and all 11 behavioural/lifestyle ERFs. The matched analysis included the four prespecified pairs only. Benjamini–Hochberg FDR correction was applied across the 36 individual pair-specific matched contrast tests and, separately, across the nine pooled four-pair matched contrasts.

# Supplementary Figure 1. Pairwise contrasts between final ERF categories.

Cell colour shows the difference between inverse-variance pooled standardised beta estimates for each ordered pair of ERF categories.

# Supplementary Figure 2. Individual schizophrenia PRS associations across all 49 ERFs.

Points show standardised beta estimates and horizontal lines show 95% confidence intervals. Colours identify the five ERF categories; filled points indicate within-PRS FDR < 0.05 and open points indicate associations that did not survive within-PRS FDR correction.

# Supplementary Figure 3. Individual results for the four primary matched comparisons.

Points show the separate perception/subjectivity and objective/non-perception estimates for each matched pair; horizontal lines show 95% confidence intervals. Circles denote perception/subjectivity measures and triangles denote objective/non-perception measures.
